# Geometry of Braided DNA Dictates Supercoiling Partition

**DOI:** 10.1101/2024.10.08.617221

**Published:** 2024-10-11

**Authors:** Yifeng Hong, Seong ha Park, Hanjie Wang, Michelle D. Wang

**Affiliations:** 1 Department of Electrical and Computer Engineering, Cornell University, Ithaca, NY 14853, USA; 2 Department of Physics & LASSP, Cornell University, Ithaca, NY 14853, USA; 3 Howard Hughes Medical Institute, Cornell University, Ithaca, NY 14853, USA

## Abstract

During DNA replication, the replisome must rotate relative to the DNA substrate, generating supercoiling that must be partitioned in front of or behind the replisome. Supercoiling partitioned behind the replisome may intertwine (or braid) daughter DNA molecules and restrict chromosome segregation. Supercoiling partitioning and torsional resistance at the replisome should depend on the geometry of the two daughter DNA molecules, determined by their end separations. However, experimental investigation of DNA braiding under well-defined DNA geometry has proven challenging. Here, we present methods to engineer braiding substrates of defined geometry, from minimal to significant end separations. We then directly measured the torque required to braid these substrates using an angular optical trap (AOT) and found that the torque required to initiate the braiding during the first 0.5 turn critically depends on the end separation. Once braiding started, we found that the subsequent effective twist persistence length of DNA braiding is about 20–30 nm, insensitive to the end separations. Our work highlights the crucial role of braiding geometry in dictating supercoiling partitioning and torque build-up during replication. It suggests that dynamic modulation of end separation on the daughter DNA molecules could serve as a mechanism to regulate replication progression *in vivo*.

During DNA replication, a replisome duplicates the parental DNA into two daughter DNA molecules. Because of DNA’s helical structure, a progressing replisome must track the DNA helical groove via rotation relative to the DNA substrate^1–5^. This rotation can potentially intertwine the two daughter molecules and thereby prevent chromosome segregation during mitosis^3, 6–12^. In addition, DNA replication inherently creates torsional stress that, in turn, hinders the progression of the replisome^1, 3–5^. *In vivo*, these difficulties are normally relieved by the action of topoisomerases. Although topoisomerases are essential in relaxing torsional stress^2, 13, 14^, they cannot always keep up with replication^12, 15–20^. Therefore, torsional stress accumulation and daughter-strand intertwining can occur during replication elongation, especially as the replisome approaches termination^15–20^. Despite this problem being inherent to replication, we have a limited understanding of the underlying mechanisms governing torsional stress accumulation and daughter-strand intertwining (or braiding).

Previously, we hypothesized that we could investigate this problem based on the torsional mechanical properties of the substrates^4^. Because torque must be balanced at the replisome, supercoiling partitioning between the front and back of the replisome is solely determined by the torsional properties of the single-DNA molecule in front of the replisome and the double-DNA molecules behind the replisome. Notably, models of the two daughter DNA molecules behind the replisome highlight that these properties critically depend on the spacing of these two molecules at each of their ends^21, 22^. *In vivo*, the end separation of the two daughter DNA molecules at the replisome might be relatively small, estimated by the size of the replisome^4, 23^, when the replisome is actively elongating. However, this separation may significantly increase when the replisome stalls under replication stress, where a host of enzymes descends onto the fork to repair and transiently reconfigure the DNA at the fork to facilitate replication restart^24–27^. At the non-replisome end, as the two daughter DNA molecules extend away from the fork, the separation can also significantly increase until structural maintenance of chromosomes (SMC) proteins bring the two strands together^28, 29^. Taken together, the separations at both ends may change throughout the course of replication elongation, dynamically modulating the torsional properties of DNA substrates, and thus the supercoiling partitioning.

Single-molecule studies of DNA braiding have also found a strong dependence of braided DNA torsional properties on DNA end separations^21, 30–33^. However, investigating DNA braiding under well-defined DNA geometry has proven challenging. Experiments using multiple optical traps can control the geometry of the two DNA molecules while braiding^34, 35^, however their end separations are limited to large distances due to the micron-sized dimensions of the trapping particles. Other studies selected braided tethers anchored to surfaces at random locations^21, 30–33, 36–44^ and estimated the anchor separation by assuming the separations at both ends were identical^21, 30–33, 38^. However, this equal-separation assumption might not be valid in practice. Importantly, torsional studies of DNA braiding require the ability to directly measure the torque to braid the DNA, which can provide an additional quantity to validate the theoretical models for braiding^21, 22, 45–54^, however direct torque measurement has been lacking in most experimental works.

In this work, we developed methods to engineer DNA braiding substrates of well-defined geometries and directly measured the torque to braid these substrates. Our measurements provide the torque required to initiate the braiding, the torsional stiffness of braided DNA, and the torque required to buckle a DNA braid into a plectoneme. The framework laid out in this work should provide insights into how braiding geometry impacts replication-generated torsional stress and the extent of the daughter-strand intertwining during replication.

## Results

### Engineered DNA Braiding Substrates

**Fig. 1.**
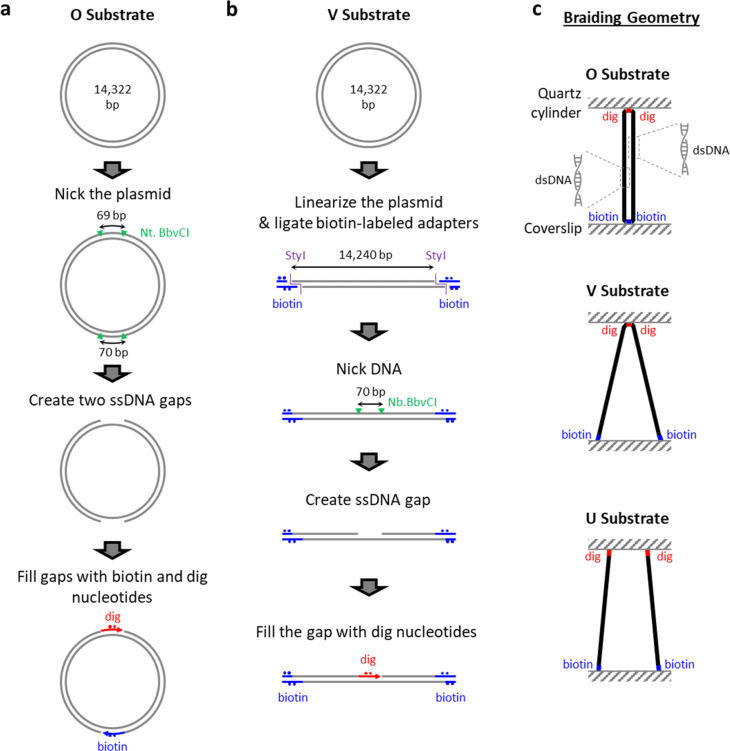
Construction of braiding substrates with defined geometries. **a.** ‘O substrate’ design and preparation. The final product has two dsDNA strands free for braiding, each with a length of 7.1 kb. The top end of the ‘O substrate’ is purely labeled with digoxigenin (red), while the bottom end is purely labeled with biotin (blue), in order to give extra-small end separations of the two ‘daughter’ DNAs once they are anchored to corresponding surfaces for single-molecule assays. **b.** ‘V substrate’ design and preparation. The final product has two dsDNA ‘daughter’ strands free for braiding, each with a length of 7.3 kb (where the uncertainty of the biotinylated anchor length is considered). The middle of the template is purely labeled with digoxigenin (red), in order to give an extra-small end separation once anchored to the surface. The two purely biotin-labeled (blue) ends allow variation of end separations once they are anchored. **c.** Three different braiding geometries, achieved by different braiding substrates.

To study DNA braiding under well-defined geometry, we developed methods to engineer two DNA braiding substrates (Methods), namely the ‘O substrate’ (Fig. 1a) and the ‘V substrate’ (Fig. 1b). These two substrates were both constructed from a customized plasmid (Methods), both with two long DNA segments having identical lengths free for braiding, as *in vivo* the leading and lagging strands are coordinately synthesized at nearly the same rate^55^.

The ‘O substrate’ was designed to have minimal end separations between the two ‘daughter’ DNA segments, each at 7.1 kb (Fig. 1a). This was achieved by creating two ssDNA gaps symmetrically on opposite sides of the plasmid, followed by filling in with digoxigenin or biotin-labeled nucleotides at these opposite sides, respectively. The final product thus has two distinct anchoring sites, each with a length of ~ 70 bp, which serves as a spacer for the end separation. In addition, the ‘O substrate’ was designed for each of the ‘daughter’ DNAs to be nicked, as the leading and lagging strand are each considered to be able to freely rotate around its helical axis during replication^1, 2, 4^.

The ‘V substrate’ was designed to have one end of the braid with a minimal separation length (≪ 7.3-kb-long ‘daughter’ DNAs for braiding), while allowing the other end to have a greater separation (Fig. 1b, Methods). To achieve this, the plasmid was linearized and then ligated with two biotin-labeled adapters. A ssDNA gap was created in the middle of the template, resulting in a constrained anchoring length of 70 bp containing digoxigenin-labeled nucleotides. The ‘V substrate’, as constructed, has one ‘daughter’ DNA nicked while the other one is not nicked. Thus, the torsionally constrained ‘daughter’ DNA requires to be nicked prior to torsional measurements to prevent torsion from accumulating within each individual ‘daughter’ DNA^4^.

Upon surface immobilization between an anti-digoxigenin-coated quartz cylinder and a streptavidin-coated coverslip surface (Methods), the two ‘daughter’ DNAs of the ‘O substrate’ have a minimal end separation at each end (Fig. 1c, top). The digoxigenin-labeled end of the ‘V substrate’ yields a small end separation when attached to an anti-digoxigenin-coated quartz cylinder while the end separation of the two biotin-labeled anchors can vary when attached to a streptavidin-coated coverslip surface. This separation can be substantial compared with the length of each ‘daughter’ DNA (Fig. 1c, middle).

In addition, to allow for a braiding geometry with wider separations at both ends, we used a more standard braiding substrate by anchoring individual 6.5 kb DNA molecules at random locations on the quartz cylinder and the coverslip surface and then selected a cylinder that was tethered to the surface via two DNA molecules^4^. This substrate allows both end separations can be substantial, compared to the length of each ‘daughter’ DNA molecule (Fig. 1c, bottom). For convenience, we call this final product the ‘U substrate’.

## Braided DNA with Minimal End Separations

**Fig. 2.**
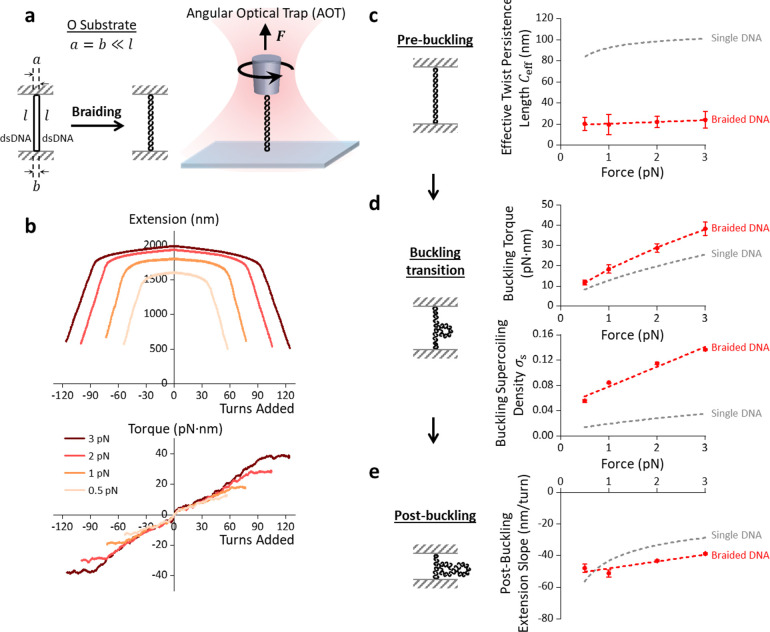
Torsional mechanical properties of braiding the ‘O substrate’. **a.** Experimental configuration for braiding the ‘O substrate’ using the AOT. The ‘O substrate’ is torsionally constrained between the coverslip and the quartz cylinder, with both anchoring lengths a and b constrained to be ~ 70 bp (Fig. 1a), significantly smaller than each individual ‘daughter’ DNA length l. Turns are added to the ‘O substrate’ by rotating the cylinder held at a constant force. **b.** Torsional measurements of braided ‘O substrate’ held at difference forces (each measurement was averaged from N=15 braided molecules). As turns were introduced, extension and torque were simultaneously measured via the AOT. **c.** Effective twist persistence length $C_{eff}$ of braided ‘O substrate’ (inset) versus force, in comparison with that of a single DNA molecule based on the Bouchiat and Mézard theory^67, 68^ (grey dashed curve). **d.** Buckling torque and buckling supercoiling density of braided ‘O substrate’ (inset) versus force, in comparison with previously measured results from a single DNA (grey dashed curves)^60^. The grey dashed line intercepts to $\sigma_{s}\;\sim \;0.05$ at zero tension, close to Marko’s prediction^69^. Here, the supercoiling density of braided DNA, alternatively the catenation density, is defined as $Ca/L_{k0}$, where Ca is the catenation number and $L_{k0}$ is the linking number of one of the ‘daughter’ DNAs for braiding. **e.** Post-buckling extension slope of braided ‘O substrate’ (inset) versus force, in comparison with previously measured results from a single DNA (grey dashed curve)^60^. In c, d, and e, each red dot represents an averaged result of N=15 molecules with error bars being standard deviations. Red dashed curves are fits to the measurements for ease of visualization.

To determine the torque required to braid the ‘O substrate’, we used the angular optical trap (AOT), which allows simultaneous measurements of torque, rotational angle, force, and displacement via a nanofabricated quartz cylinder^4, 56–62^. The ‘O substrate’ has identical end separation at both ends (a and b), which is significantly smaller than the length l of each ‘daughter’ DNA molecule (a=b≪l) (Fig. 2a). Once torsionally constrained between the coverslip surface and the cylinder (Fig. 2a, Methods), the ‘O substrate’ was held at a constant force (0.5 – 3.0 pN) and braided via rotation of the cylinder. Thus, the number of turns added via the cylinder is the same as the catenation number.

Using the AOT, we measured the torque required to braid the ‘O substrate’ while simultaneously monitoring its corresponding extension (Fig. 2b). As turns are added, the torque increases due to resistance to braiding, and the extension decreases due to the two ‘daughter’ DNA molecules helically wrapping around each other to form a braided structure (Fig. 2c). When the braiding torque reaches a critical value (Fig. 2d, top panel), the torque plateaus while the extension shortens sharply, indicating buckling of the braided DNA to form a plectoneme^63^. We found that the turns required to buckle a DNA braid depend on force, with more turns required to buckle the braid under a higher force (Fig. 2d, bottom panel). As more turns are added, the plectoneme of the braided DNA continues to extrude, indicated by a linear extension decrease (Fig. 2e)^21, 22, 30, 31^. In addition, we found that the torque-turns relation is essentially an odd function, antisymmetric about zero turn number, and the extension-turns relation is essentially an even function, symmetric about zero turn number, although small degrees of asymmetries are detected (Fig. S1; Fig. S2a) (likely due to the helix-specific interactions^47–51^). These symmetries suggest that the inherent chirality of the DNA molecules does not play a significant role in the torsional mechanical properties of braiding. As the aim of our work is to understand the supercoiling generation during replication, we therefore focus on the torsional modulus of a right-handed braid (R-braid). These behaviors are reminiscent of the torsional mechanics of a single DNA molecule^60, 64^.

We found that the torque increases with turns in a linear fashion near zero turn, albeit with a slight discontinuity, and then exhibits slight nonlinearity with increasing turns, indicating a twist-stiffening effect, as a previous theoretical work suggested^22^ (Fig. S1). To characterize the torsional resistance to braiding at a small number of turns, we determined the torsional stiffness for braiding using the slope of the torque-turns relation at the corresponding range (Methods). We then converted this stiffness to the effective twist persistence length $C_{eff}$, a parameter independent of the DNA length (Fig. 2c). We found that the $C_{eff}$ of a DNA braid is about 5 times smaller than the $C_{eff}$ of a single DNA, consistent with our previous estimation of a DNA braid^4^. Moreover, the $C_{eff}$ of a DNA braid is found to be insensitive to the tension (0.5 – 3 pN), in contrast to that of a single DNA. Thus, while the smaller $C_{eff}$ of a DNA braid reduces the torsional stress to the replication fork progression, it also partitions more supercoiling behind the replisome. These obtained results from the ‘O substrate’ will serve as a baseline for comparison with other geometries of braided DNA.

### Braided DNA with a Substantial End Separation Results in a Torque Gap

**Fig. 3.**
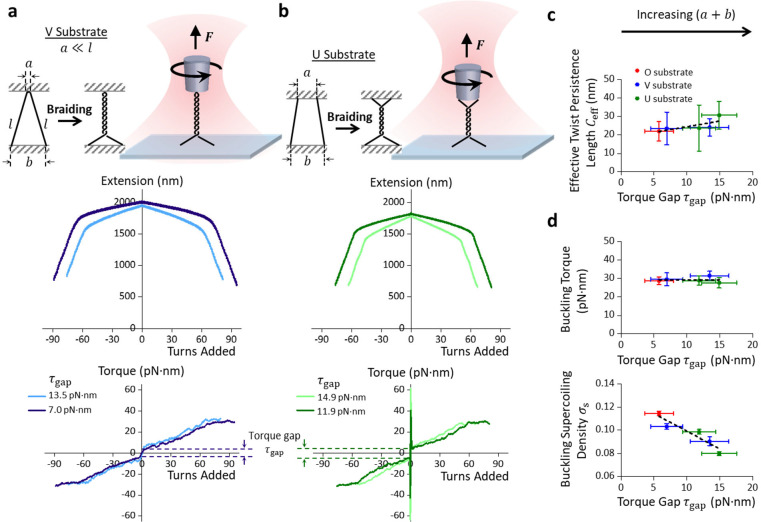
Torsional mechanical properties of braiding the ‘V substrate’ and the ‘U substrate’. **a.** Experimental configuration of braiding the ‘V substrate’ using the AOT (top panel). The ‘V substrate’ is torsionally constrained between the coverslip and the quartz cylinder, with only the top anchoring separation (at the cylinder) constrained to be ~ 70 bp while the bottom anchoring separation can vary (Fig. 1b). Torsional measurements of braided ‘V substrate’ held at 2 pN (bottom panel). The dashed lines intercept the torque gap $\tau_{gap}$ caused by nonzero net anchor separations of a+b (Methods). Data shown in dark(light) blue were data averaged from N=12(5) braided molecules. **b.** Experimental configuration of braiding the ‘U substrate’ using the AOT, where the anchor separations at both ends can vary (top panel). Torsional measurements of braided ‘U substrate’ held at 2 pN (bottom panel). Data shown in dark(light) green were averaged from N=7(11) braided molecules. **c.** Effective twist persistence length $C_{eff}$ of braided DNA versus $\tau_{gap}$ at 2 pN, characterized by a linear fit (black dashed line). **d**. Buckling torque and buckling supercoiling density of braided DNA versus $\tau_{gap}$ at 2 pN. The buckling torque is nearly constant (top black dashed line) at different $\tau_{gap}$, suggesting its insensitivity to the braiding geometry (top panel). The buckling transition of braided DNA occurs at a smaller supercoiling density as $\tau_{gap}$ increases (bottom panel), characterized by a linear fit (bottom black dashed line). In c and d, the red dots represent data measured with the ‘O substrate’ (Fig. 2b), the blue dots represent data measured with the ‘V substrate’ (Fig. 3a), and the green dots represent data measured with the ‘U substrate’ (Fig. 3b). Error bar are standard deviations.

We then carried out similar torsional measurements using the ‘V substrate’ with one end separation being significantly small (a≪l) while the other end separation (b being substantial (Fig. 3a) and the ‘U substrate’ with both end separations (a and b) being substantial (Fig. 3b). The resulting torque-turns and extension-turns relations are similar to those measured with the ‘O substrate’ (Fig. 2b) but with some significant differences. One distinguishing feature is the more significant torque discontinuity between −0.5 turn and +0.5 turn (Methods), while the extension decreases more steeply with added turns and the buckling occurs at a much smaller number of turns (Fig. 3a, Fig. 3b). In addition, torsional measurements using the ‘U substrate’ (Fig. 3b) not only reveal such a torque discontinuity but also exhibit a more dramatic torque spike near the zero turn which we will discuss in detail in the following subsection.

Here, we characterized the torque discontinuity between the −0.5 turn and +0.5 turn as the torque gap $\tau_{gap}$ (highlighted by the dashed lines in the bottom panels of Fig. 3a and Fig. 3b). According to a simple geometrical model^21^ for a DNA braid (Methods), $\tau_{gap}$ should depend on the total end separation, a+b (Fig. S3a), which also determines the DNA crossing angle to initiate the braiding. Hence, the larger the a+b, the larger the DNA crossing angle to initiate the braiding, and the larger the $\tau_{gap}$.

After carefully examining the results from all the braiding substrates (Fig. 1c), we found that the effective twist persistence length $C_{eff}$ is insensitive to $\tau_{gap}$ (Fig. 3c), likely because the measurements are still within the linear torque range (at a small number of turns). In addition, the buckling torque remains essentially the same for all the measured $\tau_{gap}$ values (Fig. 3d, top panel). This suggests that buckling occurs at a critical torque when the DNA crossing within a braid reaches a critical angle. An increased $\tau_{gap}$ thus provides a larger DNA crossing angle at the start of braiding, reducing the required turns to reach the critical buckling torque. Consistent with this interpretation, we found that the critical supercoiling density for buckling decreases with $\tau_{gap}$ (Fig. 3d, bottom panel) regardless of the braiding handedness (Fig. S2b).

Thus, we found that as the total end separation a+b becomes nonzero, a torque gap $\tau_{gap}$ appears. The presence of this torque gap indicates a stronger resistance to initiate the braiding in comparison to the subsequent addition of braiding twists. During DNA replication, such a torque gap can serve as a barrier for supercoiling partitioning behind the replisome, limiting daughter strand intertwining.

### Braided DNA with Substantial Separations at Both Ends Leads to a Torque Overshoot

**Fig. 4.**
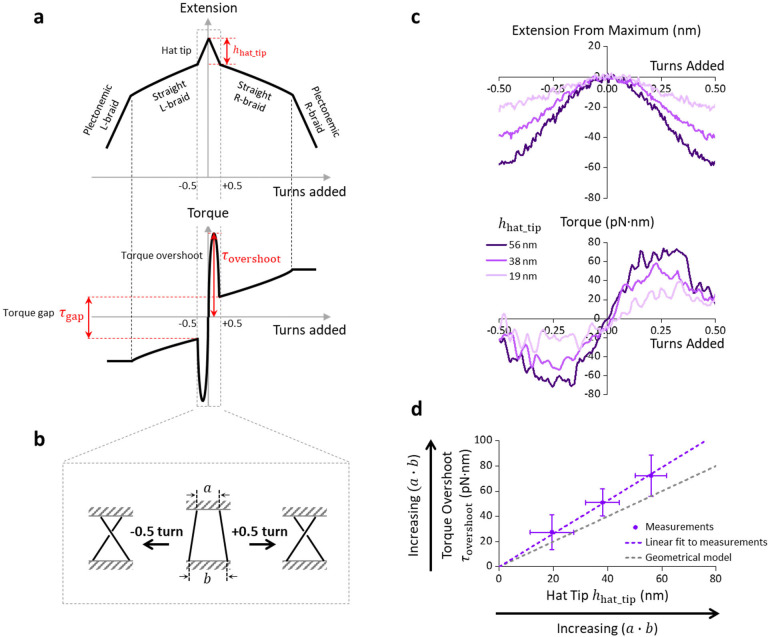
Torsional responses of rotating two DNA strands within ± 0.5 turn prior to braiding. **a.** A sketch depicting torsional responses of a double-DNA held at a constant force. From 0 turn to ±0.5 turn, a sharp decrease in extension (top panel) can occur symmetrically in the extension-turns relation (also called the hat curve). We defined the size of this decrease as $h_{hat\_tip}$, corresponding to a sinusoidal-like torque profile with its peak value defined as $\tau_{overshoot}$ (bottom panel). The magnitudes of $h_{hat\_tip}$ and $\tau_{overshoot}$ are, in theory, proportional to the product of each anchor separation a·b (Methods). As more turns are introduced, the two DNA strands will first intertwine with each other and then form plectonemic structures. **b.** Cartoon illustration of the geometry of rotating DNA strands by ±0.5 turn. **c.** Torsional measurements within ±0.5 turn using the ‘U substrate’. Shown traces were averaged from N=13,N=18,N=9 double-DNA molecules corresponding to hat tip sizes from small to large. **d**. $\tau_{overshoot}$ versus $h_{hat\_tip}$ from c. The purple dashed line indicates a linear fit to the data, whose slope is somewhat larger than the prediction (grey dashed line). Error bars are standard deviations.

In contrast to the ‘O substrate’ and the ‘V substrate’, the ‘U substrate’ can have significant end separations at both ends. We found that the most striking feature of this substrate is the torque spikes near zero turn (Fig. 4a–c). As shown in Fig. 4c that focuses on a region within ±0.5 turn, we detected two torque spikes: a positive spike centered around +0.25 turn and a negative spike centered around −0.25 turn. These spikes can be an order of magnitude greater than the torque gap. Because the spikes peak sharply above the smoother torque profile after the braiding initiates, we characterized their amplitude using the parameter “torque overshoot” $\tau_{overshoot}$ (Fig. 4a). Concurrent with the observed “torque-overshoot” is a sharp extension drop at ±0.5 turn when the two ‘daughter’ DNA molecules are first brought in contact to initiate the braiding (Fig. 4b). We refer to this extension drop as the “hat tip” $h_{hat\_tip}$, since the extension-turns relation is sometimes called a hat curve.

Overall, we found that $\tau_{overshoot}$ is proportional to $h_{hat\_tip}$ (Fig. 4d). Our measured torque overshoot is somewhat greater than the expectation based on a simple geometrical braiding model^21^ (Methods), indicating that molecule-to-molecule interaction could occur even within ±0.5 turn. Nonetheless, $\tau_{overshoot}$ and $h_{hat\_tip}$ should be proportional to the product of the end separations a·b based on the geometrical model (Fig. S3b). Consistent with the prediction by this model, we did not observe any torque overshoot when braiding the ‘O substrate’ and the ‘V substrate’ (Fig. S4), which have an extra-small end separation at least at one end.

Thus, we found that as the product of end separations a·b increases, the torque overshoot $\tau_{overshoot}$ increases. The torque overshoot can be much greater than the torque gap $\tau_{gap}$ and thereby present a stronger barrier to the initiation of braiding. Consequently, during DNA replication, torque overshoot can be highly effective at limiting supercoiling partitioning behind the replisome and thus preventing the daughter strands from intertwining.

## Discussion

Our work demonstrates that geometry plays a crucial role in the torsional mechanics of DNA braiding. We show that the end separations of the two ‘daughter’ DNA molecules significantly impact the torque required to initiate the braiding: the torque gap $\tau_{gap}$ increases with the total end separation a+b, and the torque overshoot $\tau_{overshoot}$ increases with the product of end separations a·b. These findings have significant implications for supercoiling partitioning and the torsional resistance accumulation during replication.

During replication, the supercoiling generated by the replisome progression must be distributed either behind or in front of the replisome. Supercoiling partitioning behind the replisome will intertwine (braid) the two daughter DNA molecules and must be untangled before chromosome segregation. Our work suggests that this partitioning and the torsional resistance to replication are determined by the torsional properties of the DNA substrates in the front of and behind the replisome, but the geometry of the daughter DNA molecules also serves as another critical determinant.

**Fig. 5.**
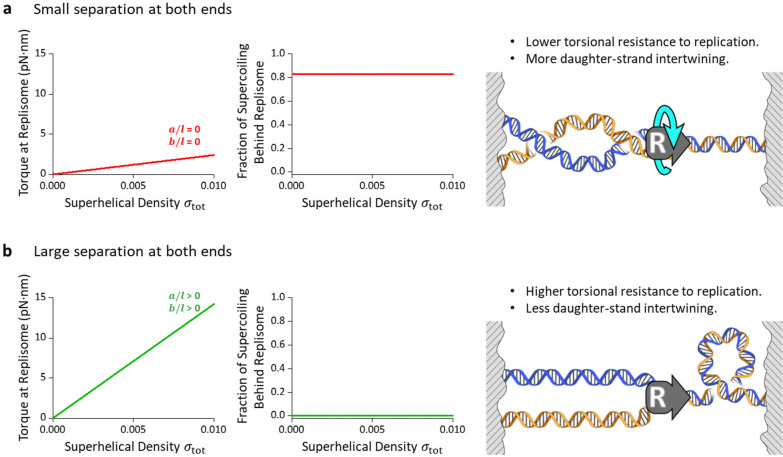
Model of supercoiling partition during DNA replication, calculated based on different braiding geometries. **a.** Braiding geometry with small separation at both ends. Calculated fraction of supercoiling partitioned to behind the replisome as a function of total superhelical density $\sigma_{tot}$ under the limit of a/l=0 and b/l=0 (middle panel), corresponding to the torsional resistance to the replisome (left panel). Under this geometrical configuration, more supercoils are absorbed to behind, intertwining daughter strands, buffering the torsional stress to replication (right panel, reprinted from^4^, with permission from Elsevier). **b.** Braiding geometry with large separation at both ends. Calculated fraction of supercoiling partitioned to behind the replisome as a function $\sigma_{tot}$ under the limit of a/l>0 and b/l>0 (middle panel), corresponding to the torsional resistance to the replisome (left panel). Under this configuration, $\tau_{overshoot}$ serves as a substantial energy barrier preventing fork rotation, effectively reducing the daughter-strand intertwining, but causing higher torsional stress to replication (right panel, reprinted from^4^, with permission from Elsevier). Parameters used for this calculation are shown in Table. S2.

For simplicity, we consider a case where DNA replication takes place over a naked DNA and proceeds to the middle of the DNA substrate. The torsional mechanics of braiding two DNA molecules measured in this work and the torsional mechanics of twisting a single DNA molecule we measured previously^4, 60, 64, 65^ can then be used to make predictions of the supercoiling partitioning and the torsional resistance to replication. When the end separations of the daughter DNA molecules are small at both ends (Fig. 5a), supercoiling will partition predominantly to behind the replisome, intertwining the two daughter DNA molecules, since the torsional stiffness of braided DNA is much smaller compared with that of a single DNA molecule. However, there is less torsional resistance for replisome progression since the braided DNA effectively buffers the torsional stress. In contrast, when the end separations of the daughter DNA molecules are large, the large torque overshoot (Fig. 5b) and torque gap (Fig. S5) impose a barrier for supercoiling partitioning to behind the replisome. Thus, supercoiling will be directed predominantly to the front of the replisome, leading to the two daughter DNA molecules minimally intertwined. However, there is significant torsional resistance for replisome progression since the single DNA is much less effective at buffering the torsional stress. Interestingly, previous studies show that DNA intertwining is reduced upon inactivation of cohesion^66^, which brings together the daughter DNA molecules, supporting the proposed model here.

Although these predictions are made for the naked DNA substrates, the framework laid out here should be general. *In vivo*, DNA is associated with bound proteins, which could significantly alter its torsional properties. Indeed, converting naked DNA to chromatin has been shown to significantly change the torsional mechanics of chromatin, which will in turn impact supercoiling partitioning^4^. Our work suggests that the geometry should also play a crucial role in supercoiling partitioning during replication on these protein-bound substrates.

## Supplementary Material

Supplement 1
