# Supplementary material for: Geometry of Braided DNA Dictates Supercoiling Partition
Source: bioRxiv. 2024 Oct 11:2024.10.08.617221. Preprint. [Version 1] doi: 10.1101/2024.10.08.617221 (PMC11482784; doi:10.1101/2024.10.08.617221)
Supplement: Supplement 1 [file media-1.pdf]

Supplementary Information for

Geometry of Braided DNA Dictates Supercoiling Partition

Yifeng Hong[1], Seong ha Park[2,3], Hanjie Wang[2], and Michelle D. Wang[2,3,*]

[1]Department of Electrical and Computer Engineering, Cornell University, Ithaca, NY 14853, USA

[2]Department of Physics & LASSP, Cornell University, Ithaca, NY 14853, USA

[3]Howard Hughes Medical Institute, Cornell University, Ithaca, NY 14853, USA

*Correspondence: mwang@physics.cornell.edu

Methods

Plasmid Cloning

The braiding templates 'O substrate' and 'V substrate', were created from pMDW168. This plasmid was constructed via Gibson assembly and site directed mutagenesis. The Gibson assembly used four dsDNA segments: two ~ 7 kb segments for the arms of the braiding substrate and two ~ 70 bp segments for the anchors. Both ~ 7 kb segments were amplified via PCR from pMDW133 using primers (Arm1 F, Arm1 R, Arm2 F, Arm2 R, Table S1) and Q5 DNAP (NEB, M0491S), followed by purification with Purelink PCR columns (Invitrogen, K310001). The ~ 70 bp anchor segments were produced by amplifying two 60 nt ssDNA (Anchor A template, Anchor T template, Table S1) oligonucleotide via PCR with primers (Anchor A_F, Anchor A_R, Anchor T_F, Anchor T_R, Table S1). The resulting two anchors and two arms were mixed in a 5:1 molar ratio and assembled using NEBuilder HiFi DNA assembly mastermix (NEB, E2621S). The assembled DNA was transformed into NEB 5α cells (NEB, C2987H) to make an intermediate plasmid. This intermediate plasmid had an unwanted Nt.BbvCI (NEB, R0632) recognition sequence on top of four that was included by design. This site was removed using site directed mutagenesis, first using PCR amplification from the intermediate plasmid with primers (KLD nickRemoval F, KLD nickRemoval R, Table S1), followed by a KLD reaction on the PCR product

with T4 PNK (NEB, M0201S), T4 ligase (NEB, M0202S) and DpnI (NEB, R0176S). We transformed the final product into NEB 5α competent cells, resulting in plasmid pMDW168.

DNA Template Construction

The preparation of 'O substrate' is shown in Fig. 1a. The plasmid pMDW168 was digested with Nt.BbvCI to introduce two pairs of nicks, each pair containing two nicks spaced ~ 70 bp apart, for a total of four nicks on the plasmid. Two ~ 70-nt ssDNA gaps were then created by incubating the nicked plasmid with excess competitor ssDNA with complementary sequence (Dig-end competitor 1 and 2, Bio-end competitor 1 and 2, Table S1) using a thermocycler and the following program: 15 min at 82 ^{o}C, then 42 cycles of 1 min with -1 ^{o}C temperature gradient per cycle. The two created ssDNA gaps were filled with digoxigenin (Roche, 11093088910) and biotin (Invitrogen, 19524016) labeled nucleotides via Hemo KlenTaq (NEB, M0332). The Hemo KlenTaq does not have strand displacement or exonuclease activity, resulting in a filled gap with an unligated nick at the end. The final product was purified with Purelink PCR purification columns. The resulting circular DNA had two torsionally unconstrained dsDNA segments free for braiding, with each end separation constrained by ~ 70 bp.

The preparation of 'V substrate' template is shown in Fig. 1b. The plasmid pMDW168 was linearized by digestion with StyI-HF (NEB, R3500). The ~ 500 bp long biotin labeled adapter was amplified via PCR from the plasmid pMDW111 with ~ 25% of the dATP replaced with biotin-dATP. The PCR was performed using Taq DNAP (NEB, M0273) and primers (0.5 kb PCR F, 0.5 kb PCR R, Table S1). The amplified adapter DNA was digested with StyI-HF to produce overhangs matching the linear template. The two digested products were ligated together with T4 ligase, and then purified with Purelink PCR purification columns. The product was nicked with Nb. BbvCI (NEB, R0631) and incubated with the competitor ssDNA (Dig-end competitor flip 1 and 2, Table S1) to create one 70-nt ssDNA gap using the following program: 15 min at 82 ^{o}C, then 42 cycles of 1 min with -1 ^{o}C temperature gradient per cycle. The ssDNA gap was then filled with digoxigenin labeled nucleotides via Hemo KlenTaq. The final product was purified

with Purelink PCR purification columns. The resulting 'V substrate' has one DNA segment torsionally constrained and the other one torsionally unconstrained.

The 6.5 kb torsionally unconstrained template was amplified via PCR from plasmid pMDW133, with primers (6.5 kb PCR F, 6.5 kb PCR R, Table S1)[1].

Fabrication of Quartz Cylinders

The quartz cylinders were fabricated out of a quartz wafer (Precision Micro Optics, PSQB-131332)[1-8] based on deep-ultraviolet-lithography-based nanofabrication process[1, 3, 5, 6]. These functionalized cylinders were covalently attached with anti-digoxigenin (Roche, 11333089001) via glutaraldehyde as a linker (Sigma, G5882).

Single-Molecule Chamber Preparation

Single-molecule sample chambers for DNA torsional studies using the AOT[9] were assembled with a nitrocellulose-coated coverslip (Fisher Scientific, 12544B) and a Windex-cleaned glass slide (Fisher Scientific, 1255010)[3, 6]. The chamber surface was functionalized with streptavidin (Agilent, SA10) conjugated to a coverslip coated with biotinylated bovine serum albumin (ThermoFisher, 29130). The functionalized chamber was passivated with β-casein (Sigma, C6905) prior to tethering DNA molecules.

For both the 'O substrate' and the 'V substrate', the prepared braiding substrate was immobilized on the functionalized coverslip surface. After flushing out free DNA within the chamber, the anti-digoxigenin coated quartz cylinders were flowed in to attach to the surface-immobilized DNA. In addition, the 'V substrate' was nicked with 30 U/mL Nt. BsmAI (NEB, R0121) in the nicking buffer (10 mM Tris pH 8.0, 10 mM NaCl, 0.5 mM $MgCl_2$, 1.5 mg/mL β-casein) after the cylinder attachment step to prevent torsion from accumulating within individual 'daughter' DNAs. For the 'U substrate' braiding assay (by 6.5 kb DNA), the DNA was

first mixed with the quartz cylinders to increase the fraction of double-DNA tethers, and then introduced to the sample chamber for surface immobilization.

Prior to torsional measurements on the AOT, each chamber was flushed with the topoisomerase reaction buffer (10 mM Tris-HCl pH 8.0, 50 mM KCl, 50 mM NaCl, 3 mM $MgCl_2$, 1 mM ATP, 0.1 mM EDTA, and 1.5 mg/mL β-casein)[3]. Unless otherwise stated, all these processes were performed at room temperature (23 ^{o}C).

Data Acquisition and Analysis

The torsional measurements for braided DNA molecules using the AOT were performed at a laser power of 40 mW entering the objective to minimize the possibility of photo-damage while preventing cylinder slippage[10, 11]. These measurements were similar to those so-called hat-curve measurements performed for a single DNA molecule[1, 3, 6, 7, 12-16]. In each measurement, the tethered quartz cylinder was held at a constant force and rotated at 2 turn/s to add or remove turns to the braiding substrate. Data of extension and torque were simultaneously measured at 10 kHz and then recorded after averaging to 400 Hz. Torque data was filtered with a 2 s sliding window to reduce the level of Brownian noise. To resolve the torque overshoot that occurs within $\pm$0.5 turn (Fig. 3b, Fig. 4c, Fig. S4), the torque filter sliding window size was reduced to 0.01 s. All the measurements were performed at room temperature (23 ^{o}C).

The effective twist persistence length $C_{\mathrm{eff}} = \frac{\partial \tau}{\partial \sigma_{\mathrm{c}}} \frac{1}{k_{\mathrm{B}} T \omega_0}$ of braided DNA was determined by linearly fitting the torque slope within $\frac{5}{L_{k0}} < \sigma_{\mathrm{c}} < 0.05$ for individual torsional measurements, where τ is the measured torque, σ_{c} is the catenation density, L_{k0} is the linking number of each 'daughter' DNA molecule, k_{B} is the Boltzmann constant, T is the temperature, and $\omega_0 = \frac{2\pi}{3.55\ \mathrm{nm}}$. The buckling transition of each braided DNA was determined by fitting the extension-turns relation with a 2-piecewise function, which consists of a quadratic region for the pre-buckling regime and a linear region for the post-buckling regime. The buckling torque was determined by averaging the torque data after the buckling transition, as the post-buckling

torque remains plateaued under our experimental condition (Fig. 2b, Fig. 3a, Fig. 3b). The hat tip size $h_{\mathrm{hat\_tip}}$ was determined by fitting the individual hat curves within $\pm$0.5 turn with equation (1). The τ_{gap} was obtained from the difference between the torque averaged from 0.5 to 10 turns and the torque averaged from −0.5 to −10 turn.

Geometrical Model for Braided DNA

To estimate how end separations of a double-DNA affects the torque gap τ_{gap} and the torque overshoot $\tau_{\mathrm{overshoot}}$, we extended the geometrical model[17] of a DNA braid to the general case with non-equal end separations. The extension z of the braiding substrate is

$$z(n) = \sqrt{l^2 - \frac{(a-b)^2}{4} - ab\sin^2 \pi|n|}, \qquad |n| < 0.5 \tag{1}$$

$$z(n) = \sqrt{l^2 - \left[\frac{a+b}{2} + \pi D\left(|n| - \frac{1}{2}\right)\right]^2}, \qquad |n| \geq 0.5 \tag{2}$$

where n is the catenation number, l is the end-to-end length of one 'daughter' DNA which is assumed inextensible, a and b are the end separations, and D is the diameter of a DNA braid. From equation (1) and equation (2), the size of the hat tip can be calculated as

$$h_{\mathrm{hat\_tip}} = |z(0) - z(0.5)| \approx \frac{ab}{2l}. \tag{3}$$

The torque can be derived from the thermodynamic Maxwell relation as

$$\tau(n) = \frac{Fab\sin(2\pi|n|)/4}{\sqrt{l^2 - \frac{(a-b)^2}{4} - ab\sin^2 \pi|n|}}, \qquad |n| < 0.5 \tag{4}$$

$$\tau(n) = \frac{FD}{2}\tan\alpha + \frac{k_{\mathrm{B}}TL_{\mathrm{p}}}{Dl}\sin^2\alpha\left[l\sin\alpha + 3\pi D\left(|n| - \frac{1}{2}\right)\right], \qquad |n| \geq 0.5 \tag{5}$$

where F is the axial stretching force, $\sin\alpha = \left[\frac{a+b}{2} + \pi D\left(|n| - \frac{1}{2}\right)\right]/l$, and L_{p} is the persistence length of a single DNA molecule.

Based on equation (4) and equation (5), we obtained the torque overshoot $\tau_{\text{overshoot}}$ as the torque value at n = 0.25 and $\tau_{\text{gap+,-}}$ as the torque values at n = $\pm$0.5, which can be simplified as (assuming $a \ll l$ and $b \ll l$)

$$\tau_{\text{overshoot}} \approx \frac{Fab}{4l}, \tag{6}$$

$$\tau_{\text{gap+,-}} = \frac{FD}{4} \frac{a+b}{\sqrt{l^2 - \frac{(a+b)^2}{4}}} + \frac{k_{\text{B}} T L_{\text{p}}}{D} \left[\frac{a+b}{2l}\right]^3. \tag{7}$$

Notably, the geometrical model assumes the torsional properties of the braided DNA to be symmetric under the addition of (+) and (-) supercoils. However, our measurements on the buckling transition show that the braided DNA indeed has a slight chirality (Fig. S2), consistent with the previous result measured by the magnetic tweezer[17]. Such a result might imply chirality in the braiding torque (meaning $\tau_{\text{gap+}}$ is not necessarily equal to $\tau_{\text{gap-}}$) which was not modeled here. Therefore, we defined the torque gap as difference between the torque in the DNA substrate at n = +0.5 and n = -0.5,

$$\tau_{\text{gap}} = \tau_{\text{gap+}} - \tau_{\text{gap-}}. \tag{8}$$

Calculation of Supercoiling Partition during Replication

DNA replication introduces (+) supercoiling in the DNA substrate as the parental DNA is unwound to produce two daughter strands. The overall supercoiling density of a replicating DNA substrate can be written as $\sigma_{\text{tot}} = \frac{\theta_{\text{behind}} + \theta_{\text{front}}}{L_{k0}}$, where θ_{behind} is the number of turns absorbed to behind, θ_{front} is the number of turns absorbed to front, and L_{k0} here is the total linking number of the template DNA before replication initiation (we used L_{k0} = (14000 / 10.5) turn in our calculation). Considering the scenario when the replisome proceeds to the midpoint of a replicating DNA substrate, we calculated the fraction of supercoiling behind the replisome $p = \frac{\theta_{\text{behind}}}{\theta_{\text{behind}} + \theta_{\text{front}}}$ as a function of σ_{tot}, and correspondingly the torque at the replisome $\tau_{\text{replisome}}$ (Fig. 5, Fig. S5), characterized by an effective torsional modulus of the coupled

braided and single DNA substrates[6]. It is worth noting that the first 0.5 turn of the DNA braiding does not scale with the DNA length to catenation density. For the case where a/l = b/l = 0, the C_{eff} of braided DNA was directly used to characterize the torsional modulus within the first 0.5 turn. For the case where a/l = 0 and $b/l > 0$, the torque was assumed to rise linearly to $\tau_{\mathrm{gap+}}$ in the first 0.5 turn. For the case where $a/l > 0$ and $b/l > 0$, the torque was given by equation (4). The detailed parameters used for this calculation are summarized in Table S2, where we considered a 2 pN force is applied to the DNA substrate.

Oligo Name	Sequence (5' → 3')
Arm1 F	CAATGGCCTGCGTATCTTCAA
Arm1 R	TCCGGAGAGTCAGCGATGTT
Arm2 F	TCACCATTACCGGATACAACC
Arm2 R	TGTTACGAAGATGGATGCCT
Anchor A template	AACAACAAACAGAAACAGACAAGAAACAAAGCAAAGAAACAAGAGAGA AAC AGAACACGA
Anchor T template	TCGTGTTCTTTGGTTCTTTGTCTTGTTTCCTTGTTTGTTCTTTCTTGCTTGTTT GTTGTT
Anchor A_F	TCAGGAACATCGCTGACTCTCCGGACCAAGGGCTGAGGAGCAACAACAAA C
Anchor A_R	TTCCGGTTGTATCCGGTAATGGTGACCAAGGCCTCAGCTCGTGTTCTG
Anchor T_F	GCCAAAGGCATCCATCTTCGTAACAGCTGAGGTCGTGTTCTTTG
Anchor T_R	CTTGTTGAAGATACGCAGGCCATTGCCTCAGCAGCAACAACAAAC
KLD nickRemoval F	TTTCAACGGCCTGCTCAA
KLD nickRemoval R	ATCAGCAAAACGGCGGTG
Dig-end competitor 1	GGAGCAACAACAAACAGAAACAGACAAGAAACAAA
Dig-end competitor 2	GCAAGAAACAAGAGAGAAACAGAACACGAGCTGA
Bio-end competitor 1	GGTCGTGTTCTTTGGTTCTTTGTCTTGTTTCCTTG
Bio-end competitor 2	TTTGTTCTTTCTTGCTTGTTTGTTGTTGCTGCTGA
0.5 kb PCR F	TTATTCCAAGGGTAAAACGACGGCCAGTG (underlined indicates the inserted StyI-HF site)
0.5 kb PCR R	GGAAACAGCTATGACCATG
Dig-end competitor flip 1	GCAGCAACAACAAACAAGCAAGAAAGAACAAACAA
Dig-end competitor flip 2	GGAAACAAGACAAAGAACCAAAGAACACGACCTCA

6.5 kb PCR F	Dig - GCCACCTGACGTCTAAGAAACCATTATTATCA
6.5 kb PCR R	Biotin - GCTGGTCCTTTCCGGCAATCAGG

Table S1. Primer sequences for plasmid cloning and DNA template construction.

	$a/l = b/l = 0$	$a/l = 0, b/l > 0$	$a/l > 0, b/l > 0$
**$\tau_{\text{overshoot}}$ (pN·nm)**	0	0	60
**$\tau_{\text{gap+}}$ (pN·nm)**	NA (as no discontinuity was considered for the first 0.5 turn)	5	NA (as no supercoils can partition to behind under the above $\tau_{\text{overshoot}}$)
**C_{eff} of a DNA braid (nm)**	20	25	
**C_{eff} of a single DNA (nm)**	98.5	98.5	98.5
**$k_{\text{B}}T$ (pN·nm)**	4.09	4.09	4.09
**Buckling torque of a single DNA (pN·nm)**	19.8	19.8	19.8

Table S2. Parameters used for calculating supercoiling partition during replisome elongation, related to Fig. 5 and Fig. S5.

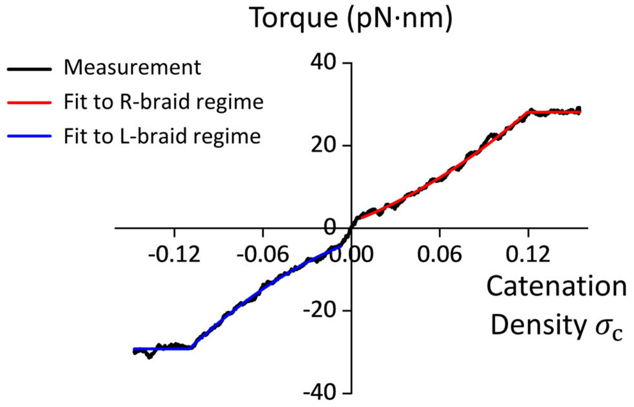

Fig. S1. Catenation-density-dependent torsional stiffness of braided DNA.

Shown in black is the measured torque versus supercoiling density from an average of *N* = 15 'O substrate' molecules held at 2 pN (same data from Fig. 2b, bottom panel). Outside of the slight discontinuity near zero, the measured torque profile can be well characterized by a two-part function, where the pre-buckling torque can be described by a 2nd order polynomial function (which demonstrates a twist-stiffening behavior[18]) and the post-buckling torque can be described by a constant.

For the R-braid, the fit (red) yields:

$\tau = [(101.3\sigma_c^2 + 18.7\sigma_c + 0.2)\ \mathrm{nm}]k_B T\omega_0$, for $\sigma_c < 0.12$

$\tau = (3.9\ \mathrm{nm})k_B T\omega_0$, for $\sigma_c \geq 0.12$

For the L-braid, the fit (blue) yields:

$\tau = [(-127.3\sigma_c^2 + 18.4\sigma_c - 0.5)\ \mathrm{nm}]k_B T\omega_0$, for $\sigma_c > -0.11$

$\tau = (-4.0\ \mathrm{nm})k_B T\omega_0$, for $\sigma_c \leq -0.11$

where $\omega_0 = \frac{2\pi}{3.55\ \mathrm{nm}}$.

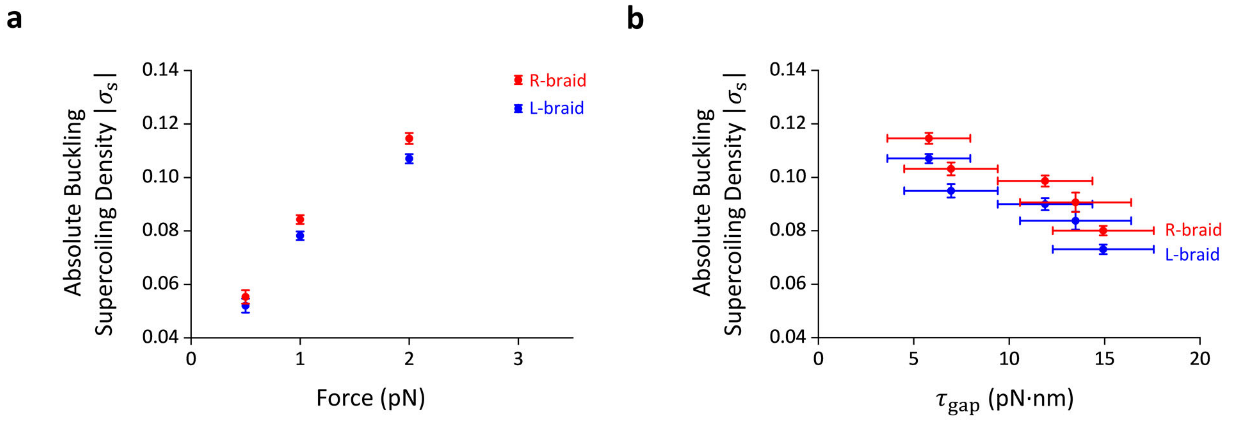

Fig. S2. Chiral buckling transition of braided DNA.

a. Chiral buckling transition of the 'O substrate' at different forces (same data set as Fig. 2b). Each measurement was averaged from *N* = 15 braided molecules. Error bars represent standard deviations.

b. Chiral buckling transition of braided DNA under different torque gaps (same data sets as Fig. 2b, Fig. 3a, and Fig. 3b). Each measurement was averaged from *N* = 15, *N* = 12, *N* = 7, *N* = 5, *N* = 11 braided molecules for τ_{gap} from small to large. Error bars represent standard deviations.

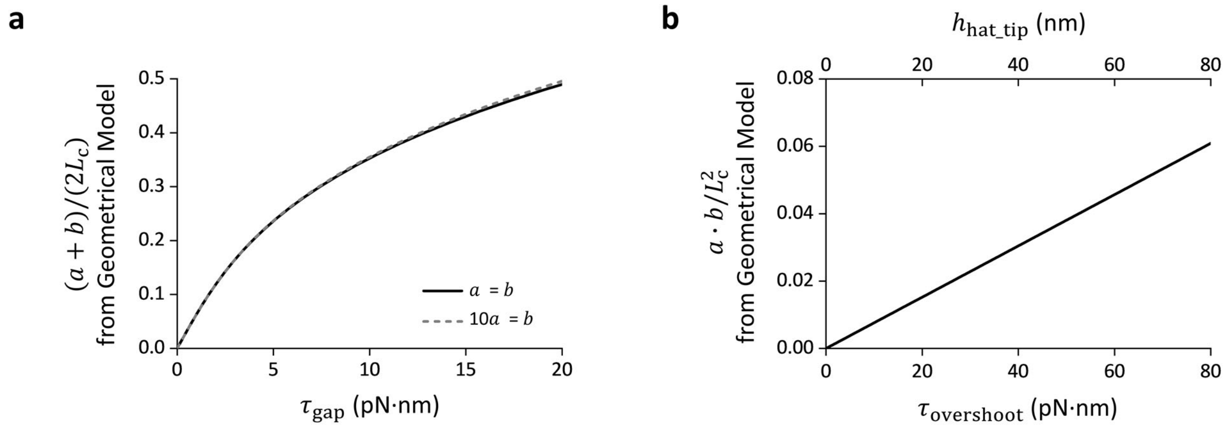

Fig. S3. General geometrical model for braided DNA. In our modeling, we used parameters: DNA persistence length L_p = 43.1 nm[19], $k_B T$ = 4.09 pN·nm, diameter of a DNA braid D = 6.5 nm[17], and F = 2 pN.

a. Estimated total end separation length normalized to the DNA contour length L_c (7 kb) from τ_{gap}, where parallel and unparallel DNA configurations give nearly the same τ_{gap}.

b. Estimated product of each end separation length normalized to the DNA contour length L_c (6.5 kb) from h_{hat_tip} and $\tau_{overshoot}$.

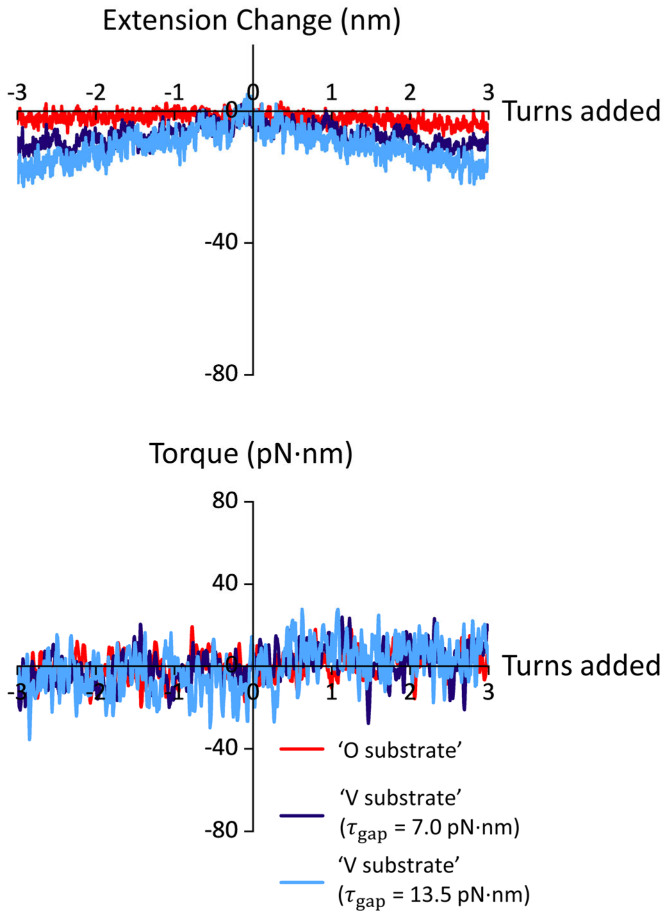

Fig. S4. Torsional measurement of braiding 'O substrate' and 'V substrate' within $\pm$3 turns.

Here, the same data sets as Fig. 2b and Fig. 3a were used. No detectable $\tau_{overshoot}$ was observed because at least one anchoring end of these braiding templates was constrained to be nearly zero.

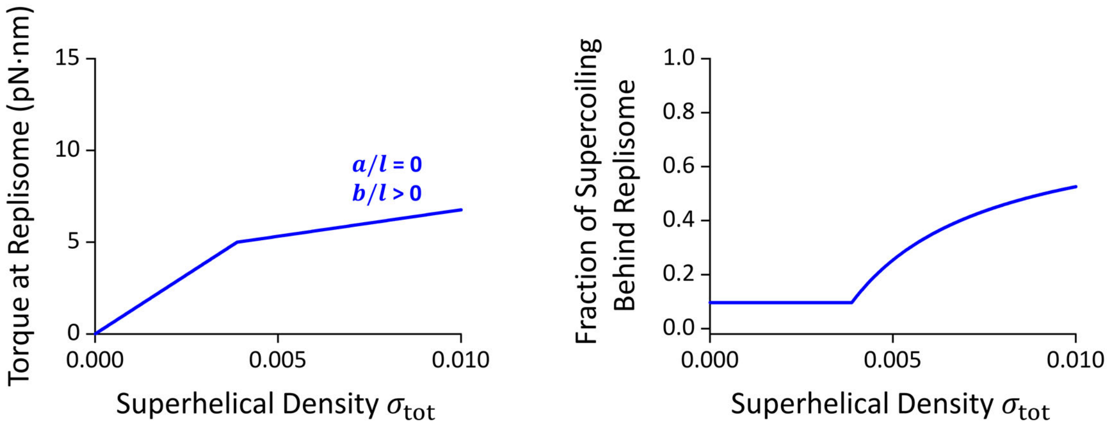

Fig. S5. Model of supercoiling partition during DNA replication, calculated based on the 'V substrate'.

Braiding geometry with a small separation at one end and a large separation at the other end. Calculated fraction of supercoiling partitioned to behind the replisome as a function of total superhelical density σ_{tot} under the limit of a/l = 0 and $b/l > 0$ (right panel), corresponding to the torsional resistance to the replisome (left panel). Under this configuration, $\tau_{\text{gap+}}$ initially serves as an intermediate energy barrier preventing fork rotation. Once the overall resistance torque exceeds $\tau_{\text{gap+}}$, more supercoils will then be absorbed behind the replisome than to the front, buffering the torsional stress to replication.

Parameters used for this calculation are shown in Table. S2.

Sequence of plasmid pMDW133

gatcctctagagtcgacctgcaggcatgcaagcttggcgtaatcatggtcatagctgtttcctgtgtgaaattgttatccgctcacaattccacacaacatacgagccggaagcataaagt
gtaaagcctggggtgcctaatgagtgagctaactcacattaattgcgttgcgctcactgcccgctttccagtcgggaaacctgtcgtgccagctgcattaatgaatcggccaacgcgcggg
gagaggcggtttgcgtattgggcgctcttccgcttcctcgctcactgactcgctgcgctcggtcgttcggctgcggcgagcggtatcagctcactcaaaggcggtaatacggttatccacag
aatcaggggataacgcaggaaagaacatgtgagcaaaaggccagcaaaaggccaggaaccgtaaaaaggccgcgttgctggcgtttttccataggctccgcccccctgacgagcatc
acaaaaatcgacgctcaagtcagaggtggcgaaacccgacaggactataaagataccaggcgtttccccctggaagctccctcgtgcgctctcctgttccgaccctgccgcttaccggat
acctgtccgcctttctcccttcgggaagcgtggcgctttctcatagctcacgctgtaggtatctcagttcggtgtaggtcgttcgctccaagctgggctgtgtgcacgaaccccccgttcagcc
cgaccgctgcgccttatccggtaactatcgtcttgagtccaacccggtaagacacgacttatcgccactggcagcagccactggtaacaggattagcagagcgaggtatgtaggcggtgc
tacagagttcttgaagtggtggcctaactacggctacactagaagaacagtatttggtatctgcgctctgctgaagccagttaccttcggaaaaagagttggtagctcttgatccggcaaa
caaaccaccgctggtagcggtggtttttttgtttgcaagcagcagattacgcgcagaaaaaaaggatctcaagaagatcctttgatcttttctacggggtctgacgctcagtggaacgaaa
actcacgttaagggattttggtcatgagattatcaaaaaggatcttcacctagatccttttaaattaaaaatgaagttttaaatcaatctaaagtatatatgagtaaacttggtctgacagtt
accaatgcttaatcagtgaggcacctatctcagcgatctgtctatttcgttcatccatagttgcctgactccccgtcgtgtagataactacgatacgggagggcttaccatctggccccagtg
ctgcaatgataccgcgagacccacgctcaccggctccagatttatcagcaataaaccagccagccggaagggccgagcgcagaagtggtcctgcaactttatccgcctccatccagtcta
ttaattgttgccgggaagctagagtaagtagttcgccagttaatagtttgcgcaacgttgttgccattgctacaggcatcgtggtgtcacgctcgtcgtttggtatggcttcattcagctccgg
ttcccaacgatcaaggcgagttacatgatcccccatgttgtgcaaaaaagcggttagctccttcggtcctccgatcgttgtcagaagtaagttggccgcagtgttatcactcatggttatgg
cagcactgcataattctcttactgtcatgccatccgtaagatgcttttctgtgactggtgagtactcaaccaagtcattctgagaatagtgtatgcggcgaccgagttgctcttgcccggcgt
caatacgggataataccgcgccacatagcagaactttaaaagtgctcatcattggaaaacgttcttcggggcgaaaactctcaaggatcttaccgctgttgagatccagttcgatgtaacc
cactcgtgcacccaactgatcttcagcatcttttactttcaccagcgtttctgggtgagcaaaaacaggaaggcaaaatgccgcaaaaaagggaataagggcgacacggaaatgttgaa
tactcatactcttcctttttcaatattattgaagcatttatcagggttattgtctcatgagcggatacatatttgaatgtatttagaaaaataaacaaataggggttccgcgcacatttccccg
aaaagtgccacctgacgtctaagaaaccattattatcatgacattaacctataaaaataggcgtatcacgaggccctttcgtctcgcgcgtttcggtgatgacggtgaaaacctctgacac
atgcagctcccggagacggtcacagcttgtctgtaagcggatgccgggagcagacaagcccgtcagggcgcgtcagcgggtgttggcgggtgtcggggctggcttaactatgcggcatc
agagcagattgtactgagagtgcaccatatgcggtgtgaaataccgcacagatgcgtaaggagaaaataccgcatcaggcgccattcgccattcaggctgcgcaactgttgggaaggg
cgatcggtgcgggcctcttcgctattacgccagctggcgaaagggggatgtgctgcaaggcgattaagttgggtaacgccagggttttcccagtcacgacgttgtaaaacgacggccagt
gaattcgagctcggtacCGGAGGATGGCAGCGTGATTTCACGGTCGAGCGTCAGCGTCCGGGTCTGGCTGTTCACCGCCAGCACACGACCACCGG
TGCTGATACCGGCATAGTCATCATCGCAGATTTCAATAACATCGCCCGGTACATGGCGAAGCCCTTCTGCGCCGACGCTGAAATCCACGGTCT
GCGTTTCCAGCAGTTCTGTTTTAATCAGCCACAGCCCGGCGCGGTGTGCCTGCCCCCGGCTGGTACAGCCAAAGGCATCCATCTTCGTAACAT
TACGACCGTAACGGGCAATGGCCTGCGTATCTTCAACAAGCTCTGTCGCCGTCTCCCAGCCGTTGTTCGGGTCAATCCAGTTCACCTCAACGG
CATTATGGCGGTCCTTCAGGGCGCTGAAGCTGTAGCGGAACGGCGCGCCATCATCCGGCATCACCACATTACTGCGGTTATAGGTCCACGTCT
TATCCGACGGTCGGTCCTGCACGAACGTCAGCGTCTGCCCGTTCCATACCGGCATACAGCGCATCGCCGAGCAGAAATCGCTGAGCACATCC
CACGCCTTACGCTGTGTGGTCAGGTACGCATTACAGGTGATGCGCGGCTCCGTGCCGCCAAAGCCGTCCGGCACTGACTGGTCGCAGTACTG
GCCGATGACATACAGCGCCCATTTATCCACATCCGCCGCACCAAGACGTTTCCCCATGCCGTAGCGCGGATGGGTCAGCATATCCCACAGACA
CCAGGCCATGTTGTTGCTGTATGCCGGTTTAAACGTTCCGTCCCAGATACCGCTGTATTGCCGCGTCTGCGGGTTATAGTTCGACGGCACCTG
CAGAATACGCCCGCGCAGATGATAATTACGGCTCACCTGCTGGCTGCCGAACTGCTCCGAGTCCACCTGCACGCCGACCAGTGCCGTGTTCG
GGTAGCACTGTTTCACATCGATGATTTCAGTGTATGACGACCAGAGCGTTTTGTTCTGCAGCTGGTCTGTGGTGCTGTCCGGCGTCATCCTGC
GCATCCGGATATTAAACGGGCGCGGCGGCAGGTTACCCATCACCACCGAGGCCAGATACTGCGAGGTGGTTTTGCCCTTAATGGTGATGTCT
TTTTCCGTCACCCAGCCACCGTTACGTTGTATCTGAACCAGCAGGCGGACTTCCGACGGATTCCTGTCACCCTTTGAGGTGGTTTCCACCAGTG
CCTGTACACCGAAGGTAAAGCGCAGACGGTCGATGTTTGCAGACGTAATGGTGCGGGTGATCGGCGTGTCATATTTCACTTCCGTACCCAGC
ACCGTCTCGGAGCCGGAGGATTCAAATCCCTCCGGCGGAGTCTGCTCCTGCTCACCAGCCCGGAACACCACCGTGACACCGGATATGTTGGT
ATTCCCCTCAGTGTCCAGCACCGGCGTACTGTTCAGCAGCACGCTTTTTAAGCCATCCACCGGACCTTCAATCGGCCCTTCGCTGATGGCATCG
ATCACACTCAGCAACTGCGTGGACTTCAGGTTGTCCTTCGCTTCGCGCGGGGTATGCCCCTTACTGCTTCCTTTACCCATTCCTCACGCTCCATA
AATGACAAAACCGCCCGCAGGCGGTTTCACATAAAACATTTTGCATCAGCGACCAATCACCACAACCTGACCACCGTCCCCTTCGTCTGCCGT
GCTGATCTCCTGAGAAACCACGCGTGACCCCACGCGCATTTCCCCGTACAGAACAGGCAGAACATTGCCCTGGGCAACCATGTTATCCAGTGA
GGAGAAATAGGTGTTCTGCTTACCGTTATCCGTTGTCTGTATACGGGGAGTTCTGGCTTTCGGTGCCAGCATCTGCGCCACACCACCGAGCAC
CATACTGGCACCGAGAGAAAACAGGATGCCGGTCATACCACCGGCCCCAATGGCTGCCCCCCATGCTGCAAGGGTGGCTCCGGCGGTAAAG
AATGATCCGGCAATGGCGGCAGCCCCCAGGACAATCTGGAATACGCCACCTGACTTGGCCCCGGCGACTCTGGGAACAATATGAATTACAGC
GCCATCAGGCAGAGTCTCATGTAACTGCGCCGTTAACCCGGACGTGCTGACGTCCCGCCCGGCAATCCGTACCTGATACCAGCCGTCGCTCAG
TTTCTGACGAAACGCCGGGAGCTGTGTGGCCAGTGCCCGGATGGCTTCAGCCCCCGTTTTCACACGAAGGTCGATGCGGCGACCAAATCGTT
GTAAATCCCCGTAAAGGCAGATGCGCGCCATGCCCGGTGACGCCAGAGGGAGTGTGTGCGTCGCTGCCATTTGTCGGTGTACCTCTCTCGTT
TGCTCAGTTGTTCAGGAATATGGTGCAGCAGCTCGCCGTCGCCGCAGTAAATTGCGGCGTGATTCGGCACTGATGAACCAAAACAGCACAGC
AGCACATCGCCCGGCTGTGCCGCTGACAACGGCACCTGATACAGCCCCGTCGCCTCCAGATTATCCAGATAGAGATTCTGGCCGTTACGCCAC
CAGTCATCCTCACGATGAAAGTCCGGCATCTCAATCCCCGCCAGATGATAAGCATCCCGGAACAGTGTGTAACAGTCCGTCACACCGTGCTCA
AAGCGCCGCCCGGTGAGATGCGGCACACAGCGGAACTTATGAATCGTCCCCCGGCAGACCAGCCACCACGGCAAATCACTCTGCACCTGCAG
CCGCCGGTCGGCCTCACTCAGCCAGGGCAGACCACCGGGGTGGCTGTGGACCAGCGCCACAATCTCACCCTGCATTTCTGCCTGCAGCCAGT
CTTCCGGCGACATACGGAAATAGCCTCCGGCTCACCGGAGATATTCACGCAGGGGAAATATCTTTCCCCCTCCGGCGTGCTTACCACGAAGCC
GCACGACTCCGCTGGCGCACATCGCCGGGCGTGCGCCAGAATCGCTGATTCTGTCTGTGTCATGGGATTTACTGCGAAAGTTTGTTAATGGA
AAGGAAGCCGCCAAAGTTGCCGACGTTATTGCGGAACTTACAACCGCTCAGGCATTTGCTGCATTTATCCTTCGTGATATCGGACGTTGGCTG
GTCATATTCATCCGCGACAGCCGGACCGCTATAACCGCACTCGTCACCGCGATAGGTCCAGGTGCAGGTGTTGGCCAGCATGATACGTCCCG

GAAAAACAGCGCCATCCGTTTCCGTCGGCGTGGACAGTACAAAGGAGGCACTCACCGCGCTCAGTTCGCTGCACTGCTCAATGCGCCAGCGG
CTGATCACCTCCTGCTCCGGATCGGCGTAACTGTTTCCGTTGACGAAGTTCACCGCATCCAGAAAACGGGCGTAAACCTTACGCCGGACCACC
GTTCCGCCGACCAGACTCTGCATATCTTCCGCCATCCCGGTGACCATACCGTACAGGTTAGAAACCGTCAGCGTGGGGCGCGTACTGGTGCCT
TTGCCATTCAGTTCAAAACCGCTCCCCTGAATGGGATACGGCTGATACTGTCGCCCCTGCCAGGTGACCGGCTCACCTTTTTCGTTCTGCTCAT
TACAGAAAAAAATAACGTTCTCCACCGACCTCTGTCAGGTCGATTTCCCAGAGCACCACGCTGGCCGACTGCTCCGCACGGGTGCATTCATTCA
GTGTTTCCTGCCGGATATCCTGCATCAGTTCACCACCTGTTCAAACTCTGCGCTGAACTCAACACGCAGCATACTGACCCGCGACGACCATTTT
GCGCAGGTCACCTTTATCTGCCGCCACTCATAAGGCGGCGTCCACAGAAAGGATTTCCAGCCCCCGTGCTCTTCCAGAAACGACTCCAGTACC
GTGGCCTCCTCACGGGGGACAGAAAGCGTCACGCTGTACGTTTTCAGGTTGGCATTCAGCCCGGCAGGCGCTCGCTGAGAATAGCCATCACC
AAAGCGCACCTTTCTTACAGAAGGGACCGAAGCCACATCCATACCGGGTTTCACTTTCCAGCGGAAGGTCTTCATCGTCCACCTCCGGAGAAC
AGGCCACCATCACGCATCTGTGTCTGAATTTCATCACGGGCACCCTTGCGGGCCATGTCATACACCGCCTTCAGAGCAGCCGGACCTATCTGC
CCGTTCGTGCCGTCGTTGTTAATCACCACATGGTTATTCTGCTCAAACGTCCCGGACGCCTGCGACCGGCTGTCTGCCATGCTGCCCGGTGTAC
CGACATAACCGCCGGTGGCATAGCCGCGCATCAGCCGGTAAAGATTCCCCACGCCAATCCGGCTGGTTGCCTCCTTCGTGAAGACAAACTCA
CCACGGTGAACAATCCCCGCTGGCTCATATTTGCCGCCGGTTCCCGTAAATCCTCCGGTTGCAAAATGGAATTTCGCCGCAGCGGCCTGAATG
GCTGTACCGCCTGACGCGGATGCGCCGCCACCAACAGCCCCGCCAATGGCGCTGCCGATACTCCCGACAATCCCCACCATTGCCTGCTTAAGC
AGAATTTCTGTCATCATGGACAGCACGGAACGGGTGAAGCTGCGCCAGTTCTGCTCACTGCCGGTCAGCATCGCCGCCATATTCTGTGCAATA
CCATCAAAGGTCTGCGTGGCTGCACTTTTTACCTGCGACATACTGTCCGTGGCGCTCTCTTCCCACTCACTCCAGCCGGACTTCAGGCCTGCCA
TCCAGTTCCCGCGAAGCTGGTCTTCAGCCGCCCAGGTCTTTTTCTGCTCTGACATGACGTTATTCAGCGCCAGCGGATTATCGCCATACTGTTC
CTTCAGGCGCTGTTCCGTGGCTTCCCGTTCTGCCTGCCGGTCAGTCAGCCCCCGGCTTTTCGCATCAATGGCGGCCCGTTTTGCCCGTTGCTGC
TGTGCGAATTTATCCGCCTGCTGCGCCAGCGCGTTCAGGCGCTCCTGATACGTAACCTTGTCGCCAAGTGCAGCCAGCTGGCGTTTGTACTCC
AGCGTCTCATCTTTATGCGCCAGCAGGGATTTCTCCTGTGCAGACAGCTGGCGACGTTGCGCCGCCTCCTCCAGTACCGCGAACTGACTCTCC
GCCTTCCACAAATCCCGGCGCTGCTGGCTGATTTTCTCATTTGCTCCGGCATGCTTCTCCAGCGTCCGGAGTTCTGCCTGAAGCGTCAGCAGG
GCAGCATGAGCACTGTCTTCCTGACGATCGCCCGCAGACACCTTCACGCTGGACTGTTTCGGCTTTTTCAGCGTCGCTTCATAATCCTTTTTCGC
CGCCGCCATCAGCGTGTTGTAATCCGCCTGCAGGATTTTCCCGTCTTTCAGTGCCTTGTTCAGTTCTTCCTGACGGGCGGTATATTTCTCCAGC
GGCGTCTGCAGCCGTTCGTAAGCCTTCTGCGCCTCTTCGGTATATTTCAGCCGTGACGCTTCGGTATCGCTCTGCTGCTGCGCATTTTTGTCCT
GTTGAGTCTGCTGCTCAGCCTTCTTTCGGGCGGCTTCAAGCGCAAGACGGGCCTTTTCACGATCATCCCAGTAACGCGCCCGCGCTTCATCGTT
AACAAAATAATCATCCTTGCGCAGATTCCAGATGTCGTCTGCTTTCTTATACGCAGCCTCTGCCTTAATCAGCATCTCCTGCGCGGTATCAGGA
CGACCAATATCCAGCACCGCATCCCACATGGATTTGAATGCCCGCGCAGTCCTGTCTGCCCAGGTCTCCAGCGTGCCCATGTTCTCTTTCAGGC
GGCGGGTCTGGTCATCAAACCCTTTCGTTGCGGCCTCGTTCGCCGCCTGCAATGCCCCGGCTTCATCGCCGGAACGCTGCAACTGAGCAACAT
ACGCAATCTGCTCCGCCGACACGTTATGGAACTGGCGAGCCATCGCCGTCAGCCCCGACGTCGGGTCTGTGGTCAGCTTCCCGAAGGCTTCA
GCGACCTTGTCCACCTCCACGCCGGATGCAGAGGAGAAACGCGCCACACTCTGGCTGATGGACGCAATCTGAGCCTCACCGCTTACCCCCGC
CTTAACCAGTGCGCTGAGTGACTCGCTGGTCTGGTTAAACGTCAGCCCTGCCGCCTGCCCGGCTCTGGACAGGACCAGCATACGATCTGCCGT
CAGTCCCGCCTGATTGCCGGAAAGGACCAGCGTTTTGTTGAAATCGGACAGGGTTGAGTTGCCCTGATACCAGGCATACGCCAGCGCACCGG
TCGCCACCGCCAGCGAGGTGGCCCCCACCATCGGCAGGGTGATCGCACCGGCAAGCCCCCTGAACATGGGGATCATCCCGCCGAAGGAGTC
CTTCACCTGCCCCCCCTGTTGCAGCAGGATCAGCCACGGACTTTGCCCGCCTGCAAGCTGCGTGGCCACGTCGGTGAACTGTGCAGGCAGCAT
ACGCATGGCGGCTTTATACTGCCCGACGGAAATCCCCGCTTTCTGTGCAGCCAGCGCCTGTCGGCTCAGCGACTGTTCAACGACTGCCGCTGT
TTTTTTCGCATCACTTTCCGTACCAGAAAAATGACGCCTGACTCTGGCCATCTGCTCGTCAAATCTGGCCGCATCCAGACTCAAATCAACGACC
AGATCGCCTACCGGTTCAGCCATACCGGACTCCTCCTGCGATCCCTTCTGATACTGTCATCAGCATTACGTCATCCTCCGTCATGTCCGCCACAT
CCGGGGAAGCGGGGATAACTTCATTCCCGTCCGGGCCAAAGCGGACACCTCCGGCAAGCCCTGCCGCTTTCTGCATCAGCACATCATCTTCA
GGCTCTTCGTCAGCCTCGCGCCGGTTCAGCAGACTGAAATCCAGCGGATGCATATCCGGATCGCTGAAAAACAGGCTGAGCACGGTGTACGT
CAGCCCGGAAAAGTGCATATCCAGCAGAACATCATGAAAATAATGGGTACTGTAAAAGCGGTGCCAGTCGGCATACTCCGTGGATGACATCC
CGGCAAGCATGGCACGCCAGTCGGGTCGCCCCATCTCACGCGCCAGTTTCAGGGCAAAACTCAGCTCACCGTCGAACACTTTCCCGCAGAAA
CAGGCTCTGCGGGCCCGGCGTCCTCTGTCTGTTCAGGGGCATTATTCACCACAAACTCATACATACCAGACAGCCGGTACACCACGTTTTCAG
CATGAGAAATTGCCTCCGTGGGCCAGGTGGTAAGCACTTCCTGCTCAATCTGTTTAACGGCTTCATTCATGGACGGCATCTGCGTCTTCTGCG
GATGGTTATGCCACAGGGACATCGCCACCAGAAACGCGCCGGTTCTGATGGCGTCTTCCACAGTAAACTTCCGGTTGCTGTCTGACTCCGCCT
GTTCTGCCTGCCGTTTCATCAGGGCGAGATGCTCAATGCGCTGCAGGGCTGACAGTTCAGAAAGCGTGACGGTCACACCGTTATGTTCAAAT
GATTCGGTTTTCAGGAACATCGCTGACTCTCCGGATTAACTGGCGGTGACGGTAATTTCTGCAACCGCAGCAAACTCACCATTACCGGATACA
ACCGGAATGTTGACCTTGCCTGCAGCAACGCCGTTCACGGTGATGGTCATACCACTGACCGACACGGTGGCTTTTGTTTTATCCGCAGACACC
GCACGAAAGCTCTTGTCGGTTACGCCCTCCGGCTGGAAGGCCACGGTCAGCGTGGTGCTCTGCCCCTTTCACCACCGAGGTGCTGGCAGGCGT
CACGGTCATGCCGGTTGCCGCTGTTACCGTGCTGCGATCTTCTGCCATCGACGGACGTCCCACATTGGTGACTTTCACCGTGCGGGTGATCAC
TTCCTTCGCCGTCACCGCCTTACCGATACTGCTGACCCAGCCACGGAACACATCGACCGTGCCGTTCGGGAAGCGGATTTTATAGGCACGGGT
ATCGCCTTCATTAAACCACGCCAGCAGCGCCTGCTGCCCCTGCTCTCCGGGCATCCACGCCAGCGTGAAGCTGGTATCTCCGGCAGATTTCTG
CCCCTGCCCGGTCGCAGTCCAGTCTGCATCTTCATCATCGAGATAGCTGTCGTCATAGGACTCAGCGGTCAGTTCGCCGGGCGTCAGGTCTTT
AACTTTTGCCAGACGCGACCAGTCAACGTCTGAAAGCGGATTCGCGTAAGGGTCACCGCTCCCCTTATAAACCCACAGGGTGGTCCCGGCAC
CTTTCACCGGCATTGTAGGATTTGGTACAGGCATAGCGTCCTCACATTTCATAGGTAATGACATAAGTCAGATCGGCTGAACTCCACAAGCCC
GCATCATCGTCGCGCCGGTAGTCATAGCCGCTGGCCACCATACTGGTGATCAAATCTGACAGTGCCGGGATATCGCTCATCACCGGATAAATC
CGGGACTCCATCCACGCATCCAGCTCTGAATCCGGCACCTGAGCAGGCAGGAAAACTTCGATATGCAGCTCCGCCTGCCAGGTATCGCTGTCC
AGCTCTTCGCCCGTGTATTCAGCGCCGGTGAGATAAACGGCAACTGCCGGAAAATCCGCCTCATCAAAAACAGCGGGGCGACCATCAAAAAA
CGTCGCCCCGGTGTCATGCTTCTCCAGTGCATCCAGTACGGCTGCACGGAGTTCAGTATGTTTCATCGCTTTATTACCATCCTCAGTTGATGCT

GCAGCGCATAGCCCAGCTCTTTCGGAAGACGTTCACGCCGTATCCGCTCAATATTTTGTTTAAACGCCGTGGTCAGCGGCACCGCCATCGGGA
TTTTCACCACATCAATGGGGTAACGGTTTTTCCCAGCCACACGCTGCATGACATGCCACCGGCCATTTTTCAGTTGCTGAATAAACGCGCCGG
GAATACGACGGTTACCCACCACAAGCACGCTGCCGCCACCTTTCAGGGATGAACGCTGCCCCTTTTTACGACGCCTGCGGCGCGAAAGGACA
ACCCGCGCATTACCCAGCTTGATTACGGGCAAATCCCCCCGGTTAACTTTGATTCTGGCCTGCGGATTTTTGACCGTGGCCCTTTTCAGCCTGG
CCCTTTCCTTTACCAGTTTCCGGCGTACCTTTGTCTCACGGGCAACCTGTGACGCCGACTGCGATATCGCGGATGAAGCAACGCGGTTAATGG
CCATTGCGGCGGCACCAGGCACCGCCGTTTTGCTGATACGGCTGAGGTTTTCAACGGCCTGCTCAAGACCTTTTATGGCCATACATCCCCCTTT
CAGCGGCGACGGTTAACGGCAGGCGGTACGCCCCGTCCAAGCCAGAGATGACAACTTCCGCCATCATCCGGCGAAACCCGATCTACCCAGAA
ATTTTCCTCACCGATGGTCAGCGTGTCTCCACGCCGCAGCTGCCGCACCTCATCAGTCCGGACAAACAGGGACGGGCTGGAGCCTTCAACGC
GCACGCCCTGTCCGGCATAGCTGATATTTTCAGGGTCATCAAAAACACCACGTATCACCGCACCTGACTGCTCACCGGATGTAATGGTGGCTG
ACGTTCCCATGTACCCGCGTATCGTTTCATCGGCGCGGGCAATGGCAGCATCGAACAGGTTATCGAAATCAGCCACAGCGCCTCCCGTTATTG
CATTCTGGCCAGGCCGCGCTCTGTCATTTCGGCTGCCACACCGGCAGAGACACGAAACGCCGTTCCCGGCAGCACAAATGCCACAGGTTCAT
CCCGCGTGGCGTGAAGTGCATCAGTATGCAGCTTCACCAGTGCCACGACCGTGACCAGTTCAGACGTATCCAGAATCACGGTATCCGGCTGC
GCTGATCCCACCTCATTTTCATGTCCGGTCAGCACATTTTCCCGGCTGAGAGGGGTGTCCTGACCGGCAGTTTCATCCGTGTCATCAAGCTCCT
CTTTCAGCTCTGCCACACGGAGCGCCAGTTCTTCTTTCGTCCCCGTCAGGCTGACATCACGGTTCAGTTGTTCACCCAGCGAGCGGAGACGGG
CAATCAGTTCATCTTTCGTCATGGACTCCTCCACAGAGAAACAATGGCCCCGAAGGGCCATGATTACGCCAGTTGTACGGACACGAACTCATC
AGGGTCAGCCAGCAGCATCAGCGGTGCTGACTGAATCATGGTGAACTCACGCGCCGGATCGCCGGTGGTCACCCAGTTTTTCGGGTAACGG
GCAGAGGCGTTAATGCCTTCGCGCTGTGCGTCCGCATCCTGAATGCAGCCATAGGTGCGCAGACCGCGTGCCTGAGTGTTCCCCAGCACCAT
CGTGTTGTCCGGCAGGAAGTTCTTTTTGACGCCGTTTTCCACGTACTGTCCGGAATACACGACGATGGCCACATCGCCATACATCCCCTTATAG
GACACCGCTTTGCCCAGGTCTTTCACCGCTGTCTCCAGCTCGGAATTAGAGCCACGACGGGTATCCAGCTTCTCCTTGACGGCTTTGAAGGAA
CGGAACAGCGCCCAGCCTTTCGGATCGAACACGATGATATTCACCACACCGCTGGCGTTCAGCGCGTAGGCTTCGATATCGTCGGTCGGGTC
ATACGTGGACTTGTCACGCTTGCTCCACTCCGTGCCGCCGGACTGCGTGATGTTATTCTCCTCACTGCGGCCCATATCCACCTCAACCGGATCG
AAGGCTTCACCGGTCATGGTGTATTTGCCCTTAAGCACGGCAGAAACTGCCTGCATCTCTTCGACCTGAGCAATGGCCAGCTCTTCGTCACGC
ATGTTCTGCATGATGATGCGACGGCGGCGGTAAGCCGGGTCCGCCAGATTCTGCGGATCTTCATCCGGCAGGCGACGCAGGGTCATCTGCG
GATTCACTTCATGCTTCGGCTTGACATATCCCGGCGTAAATTCAGAGGTGGAGCCGCCACGGGAACGGATAACCTCACCGGAAACAATCGGC
GAAACGTACAGCGCCATGTTTACCAGTCCCGGAATTTGTGAGAGATAGACTTTCTCCGTGGTGAAGGGATAGCTCTCACGGAAAAAGAGACG
CAGAAACAGCGGATCAAACTTAAATTTCTGCTCATTTGCCGCCAGCAGTTGGGCGGTTGTGTACATCGACATAAAAAAATCCCGTAAAAAAAG
CCGCACAGGCGGCCTTTAGTGATGAAGGGTAAAGTTAAACGATGCTGATTGCCGTTCCGGCAAACGCGGTCCGTTTTTTCGTCTCGTCGCTGG
CAGCCTCCGGCCAGAGCACATCCTCATAACGGAACGTGCCGGACTTGTAGAACGTCAGCGTGGTGCTGGTCTGGTCAGCAGCAACCGCAAG
AATGCCAACGGCAGCACCGTCGGTGGTGCCATCCCACGCAACCAGCTTACGGCTGGAGGTGTCCAGCATCAGCGGGGTCATTGCAGGCGCTT
TCGCACTCAATCCGCCGGGCGCGGTTGCGGTATGAGCCGGGTCACTGTTGCCCTGCGGCTGGTAATGGGTAAAGGTTTCTTTGCTCGTCATA
AACATCCCTTACACTGGTGTGTTCAGCAAATCGTTAACGGCATCAGATGCCGGGTTACCTGCAGCCAGCGGTGCCGGTGCCCCCTGCATCAGA
CGATCCAGCGCAGTGTCACTGCGCGCCTGTGCACTCTGTGGTGCTGCGGCCAGAATGCGGCGGGCCGTTTTCACGGTCATACCGGGGGTTTC
TGCCAGCACGCGTGCCTGTTCTTCGCGTCCGTGAGCCTCCTCACAGTTGAG

Sequence of plasmid pMDW168

AAAAAAACAGCGGCAGTCGTTGAACAGTCGCTGAGCCGACAGGCGCTGGCTGCACAGAAAGCGGGGATTTCCGTCGGGCAGTATAAAGCC
GCCATGCGTATGCTGCCTGCACAGTTCACCGACGTGGCCACGCAGCTTGCAGGCGGGCAAAGTCCGTGGCTGATCCTGCTGCAACAGGGGG
GGCAGGTGAAGGACTCCTTCGGCGGGATGATCCCCATGTTCAGGGGGCTTGCCGGTGCGATCACCCTGCCGATGGTGGGGGCCACCTCGCT
GGCGGTGGCGACCGGTGCGCTGGCGTATGCCTGGTATCAGGGCAACTCAACCCTGTCCGATTTCAACAAAACGCTGGTCCTTTCCGGCAATC
AGGCGGGACTGACGGCAGATCGTATGCTGGTCCTGTCCAGAGCCGGGCAGGCGGCAGGGCTGACGTTTAACCAGACCAGCGAGTCACTCAG
CGCACTGGTTAAGGCGGGGGTAAGCGGTGAGGCTCAGATTGCGTCCATCAGCCAGAGTGTGGCGCGTTTCTCCTCTGCATCCGGCGTGGAG
GTGGACAAGGTCGCTGAAGCCTTCGGGAAGCTGACCACAGACCCGACGTCGGGGCTGACGGCGATGGCTCGCCAGTTCCATAACGTGTCGG
CGGAGCAGATTGCGTATGTTGCTCAGTTGCAGCGTTCCGGCGATGAAGCCGGGGCATTGCAGGCGGCGAACGAGGCCGCAACGAAAGGGT
TTGATGACCAGACCCGCCGCCTGAAAGAGAACATGGGCACGCTGGAGACCTGGGCAGACAGGACTGCGCGGGCATTCAAATCCATGTGGGA
TGCGGTGCTGGATATTGGTCGTCCTGATACCGCGCAGGAGATGCTGATTAAGGCAGAGGCTGCGTATAAGAAAGCAGACGACATCTGGAAT
CTGCGCAAGGATGATTATTTTGTTAACGATGAAGCGCGGGCGCGTTACTGGGATGATCGTGAAAAGGCCCGTCTTGCGCTTGAAGCCGCCCG
AAAGAAGGCTGAGCAGCAGACTCAACAGGACAAAAATGCGCAGCAGCAGAGCGATACCGAAGCGTCACGGCTGAAATATACCGAAGAGGC
GCAGAAGGCTTACGAACGGCTGCAGACGCCGCTGGAGAAATATACCGCCCGTCAGGAAGAACTGAACAAGGCACTGAAAGACGGGAAAAT
CCTGCAGGCGGATTACAACACGCTGATGGCGGCGGCGAAAAAGGATTATGAAGCGACGCTGAAAAAGCCGAAACAGTCCAGCGTGAAGGT
GTCTGCGGGCGATCGTCAGGAAGACAGTGCTCATGCTGCCCTGCTGACGCTTCAGGCAGAACTCCGGACGCTGGAGAAGCATGCCGGAGCA
AATGAGAAAATCAGCCAGCAGCGCCGGGATTTGTGGAAGGCGGAGAGTCAGTTCGCGGTACTGGAGGAGGCGGCGCAACGTCGCCAGCTG
TCTGCACAGGAGAAATCCCTGCTGGCGCATAAAGATGAGACGCTGGAGTACAAACGCCAGCTGGCTGCACTTGGCGACAAGGTTACGTATCA
GGAGCGCCTGAACGCGCTGGCGCAGCAGGCGGATAAATTCGCACAGCAGCAACGGGCAAAACGGGCCGCCATTGATGCGAAAAGCCGGGG
GCTGACTGACCGGCAGGCAGAACGGGAAGCCACGGAACAGCGCCTGAAGGAACAGTATGGCGATAATCCGCTGGCGCTGAATAACGTCAT
GTCAGAGCAGAAAAAGACCTGGGCGGCTGAAGACCAGCTTCGCGGGAACTGGATGGCAGGCCTGAAGTCCGGCTGGAGTGAGTGGGAAG
AGAGCGCCACGGACAGTATGTCGCAGGTAAAAAGTGCAGCCACGCAGACCTTTGATGGTATTGCACAGAATATGGCGGCGATGCTGACCGG
CAGTGAGCAGAACTGGCGCAGCTTCACCCGTTCCGTGCTGTCCATGATGACAGAAATTCTGCTTAAGCAGGCAATGGTGGGGATTGTCGGGA
GTATCGGCAGCGCCATTGGCGGGGCTGTTGGTGGCGGCGCATCCGCGTCAGGCGGTACAGCCATTCAGGCCGCTGCGGCGAAATTCCATTTT
GCAACCGGAGGATTTACGGGAACCGGCGGCAAATATGAGCCAGCGGGGATTGTTCACCGTGGTGAGTTTGTCTTCACGAAGGAGGCAACCA
GCCGGATTGGCGTGGGGAATCTTTACCGGCTGATGCGCGGCTATGCCACCGGCGGTTATGTCGGTACACCGGGCAGCATGGCAGACAGCCG
GTCGCAGGCGTCCGGGACGTTTGAGCAGAATAACCATGTGGTGATTAACAACGACGGCACGAACGGGCAGATAGGTCCGGCTGCTCTGAAG
GCGGTGTATGACATGGCCCGCAAGGGTGCCCGTGATGAAATTCAGACACAGATGCGTGATGGTGGCCTGTTCTCCGGAGGTGGACGATGAA
GACCTTCCGCTGGAAAGTGAAACCCGGTATGGATGTGGCTTCGGTCCCTTCTGTAAGAAAGGTGCGCTTTGGTGATGGCTATTCTCAGCGAG
CGCCTGCCGGGCTGAATGCCAACCTGAAAACGTACAGCGTGACGCTTTCTGTCCCCCGTGAGGAGGCCACGGTACTGGAGTCGTTTCTGGAA
GAGCACGGGGGCTGGAAATCCTTTCTGTGGACGCCGCCTTATGAGTGGCGGCAGATAAAGGTGACCTGCGCAAAATGGTCGTCGCGGGTCA
GTATGCTGCGTGTTGAGTTCAGCGCAGAGTTTGAACAGGTGGTGAACTGATGCAGGATATCCGGCAGGAAACACTGAATGAATGCACCCGT
GCGGAGCAGTCGGCCAGCGTGGTGCTCTGGGAAATCGACCTGACAGAGGTCGGTGGAGAACGTTATTTTTTCTGTAATGAGCAGAACGAAA
AAGGTGAGCCGGTCACCTGGCAGGGGCGACAGTATCAGCCGTATCCCATTCAGGGGAGCGGTTTTGAACTGAATGGCAAAGGCACCAGTAC
GCGCCCCACGCTGACGGTTTCTAACCTGTACGGTATGGTCACCGGGATGGCGGAAGATATGCAGAGTCTGGTCGGCGGAACGGTGGTCCGG
CGTAAGGTTTACGCCCGTTTTCTGGATGCGGTGAACTTCGTCAACGGAAACAGTTACGCCGATCCGGAGCAGGAGGTGATCAGCCGCTGGCG
CATTGAGCAGTGCAGCGAACTGAGCGCGGTGAGTGCCTCCTTTGTACTGTCCACGCCGACGGAAACGGATGGCGCTGTTTTTCCGGGACGTA
TCATGCTGGCCAACACCTGCACCTGGACCTATCGCGGTGACGAGTGCGGTTATAGCGGTCCGGCTGTCGCGGATGAATATGACCAGCCAACG
TCCGATATCACGAAGGATAAATGCAGCAAATGCCTGAGCGGTTGTAAGTTCCGCAATAACGTCGGCAACTTTGGCGGCTTCCTTTCCATTAAC
AAACTTTCGCAGTAAATCCCATGACACAGACAGAATCAGCGATTCTGGCGCACGCCCGGCGATGTGCGCCAGCGGAGTCGTGCGGCTTCGTG
GTAAGCACGCCGGAGGGGGAAAGATATTTCCCCTGCGTGAATATCTCCGGTGAGCCGGAGGCGTATTTCCGTATGTCGCCGGAAGACTGGC
TGCAGGCAGAAATGCAGGGTGAGATTGTGGCGCTGGTCCACAGCCACCCCGGTGGTCTGCCCTGGCTGAGTGAGGCCGACCGGCGGCTGCA
GGTGCAGAGTGATTTGCCGTGGTGGCTGGTCTGCCGGGGGACGATTCATAAGTTCCGCTGTGTGCCGCATCTCACCGGGCGGCGCTTTGAGC
ACGGTGTGACGGACTGTTACACACTGTTCCGGGATGCTTATCATCTGGCGGGGATTGAGATGCCGGACTTTCATCGTGAGGATGACTGGTGG
CGTAACGGCCAGAATCTCTATCTGGATAATCTGGAGGCGACGGGGCTGTATCAGGTGCCGTTGTCAGCGGCACAGCCGGGCGATGTGCTGC
TGTGCTGTTTTGGTTCATCAGTGCCGAATCACGCCGCAATTTACTGCGGCGACGGCGAGCTGCTGCACCATATTCCTGAACAACTGAGCAAAC
GAGAGAGGTACACCGACAAATGGCAGCGACGCACACACTCCCTCTGGCGTCACCGGGCATGGCGCGCATCTGCCTTTACGGGGATTTACAAC
GATTTGGTCGCCGCATCGACCTTCGTGTGAAAACGGGGGCTGAAGCCATCCGGGCACTGGCCACACAGCTCCCGGCGTTTCGTCAGAAACTG
AGCGACGGCTGGTATCAGGTACGGATTGCCGGGCGGGACGTCAGCACGTCCGGGTTAACGGCGCAGTTACATGAGACTCTGCCTGATGGCG
CTGTAATTCATATTGTTCCCAGAGTCGCCGGGGCCAAGTCAGGTGGCGTATTCCAGATTGTCCTGGGGGCTGCCGCCATTGCCGGATCATTCT
TTACCGCCGGAGCCACCCTTGCAGCATGGGGGGCAGCCATTGGGGCCGGTGGTATGACCGGCATCCTGTTTTCTCTCGGTGCCAGTATGGTG
CTCGGTGGTGTGGCGCAGATGCTGGCACCGAAAGCCAGAACTCCCCGTATACAGACAACGGATAACGGTAAGCAGAACACCTATTTCTCCTC
ACTGGATAACATGGTTGCCCAGGGCAATGTTCTGCCTGTTCTGTACGGGGAAATGCGCGTGGGGTCACGCGTGGTTTCTCAGGAGATCAGCA
CGGCAGACGAAGGGGACGGTGGTCAGGTTGTGGTGATTGGTCGCTGATGCAAAATGTTTTATGTGAAACCGCCTGCGGGCGGTTTTGTCATT
TATGGAGCGTGAGGAATGGGTAAAGGAAGCAGTAAGGGGCATACCCCGCGCGAAGCGAAGGACAACCTGAAGTCCACGCAGTTGCTGAGT
GTGATCGATGCCATCAGCGAAGGGCCGATTGAAGGTCCGGTGGATGGCTTAAAAAGCGTGCTGCTGAACAGTACGCCGGTGCTGGACACTG
AGGGGAATACCAACATATCCGGTGTCACGGTGGTGTTCCGGGCTGGTGAGCAGGAGCAGACTCCGCCGGAGGGATTTGAATCCTCCGGCTC

CGAGACGGTGCTGGGTACGGAAGTGAAATATGACACGCCGATCACCCGCACCATTACGTCTGCAAACATCGACCGTCTGCGCTTTACCTTCG
GTGTACAGGCACTGGTGGAAACCACCTCAAAGGGTGACAGGAATCCGTCGGAAGTCCGCCTGCTGGTTCAGATACAACGTAACGGTGGCTG
GGTGACGGAAAAAGACATCACCATTAAGGGCAAAACCACCTCGCAGTATCTGGCCTCGGTGGTGATGGGTAACCTGCCGCCGCGCCCGTTTA
ATATCCGGATGCGCAGGATGACGCCGGACAGCACCACAGACCAGCTGCAGAACAAAACGCTCTGGTCGTCATACACTGAAATCATCGATGTG
AAACAGTGCTACCCGAACACGGCACTGGTCGGCGTGCAGGTGGACTCGGAGCAGTTCGGCAGCCAGCAGGTGAGCCGTAATTATCATCTGC
GCGGGCGTATTCTGCAGGTGCCGTCGAACTATAACCCGCAGACGCGGCAATACAGCGGTATCTGGGACGGAACGTTTAAACCGGCATACAG
CAACAACATGGCCTGGTGTCTGTGGGATATGCTGACCCATCCGCGCTACGGCATGGGGAAACGTCTTGGTGCGGCGGATGTGGATAAATGG
GCGCTGTATGTCATCGGCCAGTACTGCGACCAGTCAGTGCCGGACGGCTTTGGCGGCACGGAGCCGCGCATCACCTGTAATGCGTACCTGAC
CACACAGCGTAAGGCGTGGGATGTGCTCAGCGATTTCTGCTCGGCGATGCGCTGTATGCCGGTATGGAACGGGCAGACGCTGACGTTCGTG
CAGGACCGACCGTCGGATAAGACGTGGACCTATAACCGCAGTAATGTGGTGATGCCGGATGATGGCGCGCCGTTCCGCTACAGCTTCAGCG
CCCTGAAGGACCGCCATAATGCCGTTGAGGTGAACTGGATTGACCCGAACAACGGCTGGGAGACGGCGACAGAGCTTGTTGAAGATACGCA
GGCCATTGCCTCAGCAGCAACAACAAACAAGCAAGAAAGAACAAACAAGGAAACAAGACAAAGAACCAAAGAACACGACCTCAGCTGTTAC
GAAGATGGATGCCTTTGGCTGTACCAGCCGGGGGCAGGCACACCGCGCCGGGCTGTGGCTGATTAAAACAGAACTGCTGGAAACGCAGACC
GTGGATTTCAGCGTCGGCGCAGAAGGGCTTCGCCATGTACCGGGCGATGTTATTGAAATCTGCGATGATGACTATGCCGGTATCAGCACCGG
TGGTCGTGTGCTGGCGGTGAACAGCCAGACCCGGACGCTGACGCTCGACCGTGAAATCACGCTGCCATCCTCCGGTACCGAGCTCGAATTCA
CTGGCCGTCGTTTTACAACGTCGTGACTGGGAAAACCCTGGCGTTACCCAACTTAATCGCCTTGCAGCACATCCCCCTTTCGCCAGCTGGCGTA
ATAGCGAAGAGGCCCGCACCGATCGCCCTTCCCAACAGTTGCGCAGCCTGAATGGCGAATGGCGCCTGATGCGGTATTTTCTCCTTACGCATC
TGTGCGGTATTTCACACCGCATATGGTGCACTCTCAGTACAATCTGCTCTGATGCCGCATAGTTAAGCCAGCCCCGACACCCGCCAACACCCG
CTGACGCGCCCTGACGGGCTTGTCTGCTCCCGGCATCCGCTTACAGACAAGCTGTGACCGTCTCCGGGAGCTGCATGTGTCAGAGGTTTTCAC
CGTCATCACCGAAACGCGCGAGACGAAAGGGCCTCGTGATACGCCTATTTTTATAGGTTAATGTCATGATAATAATGGTTTCTTAGACGTCAG
GTGGCACTTTTCGGGGAAATGTGCGCGGAACCCCTATTTGTTTATTTTTCTAAATACATTCAAATATGTATCCGCTCATGAGACAATAACCCTG
ATAAATGCTTCAATAATATTGAAAAAGGAAGAGTATGAGTATTCAACATTTCCGTGTCGCCCTTATTCCCTTTTTTGCGGCATTTTGCCTTCCTG
TTTTTGCTCACCCAGAAACGCTGGTGAAAGTAAAAGATGCTGAAGATCAGTTGGGTGCACGAGTGGGTTACATCGAACTGGATCTCAACAGC
GGTAAGATCCTTGAGAGTTTTCGCCCCGAAGAACGTTTTCCAATGATGAGCACTTTTAAAGTTCTGCTATGTGGCGCGGTATTATCCCGTATTG
ACGCCGGGCAAGAGCAACTCGGTCGCCGCATACACTATTCTCAGAATGACTTGGTTGAGTACTCACCAGTCACAGAAAAGCATCTTACGGAT
GGCATGACAGTAAGAGAATTATGCAGTGCTGCCATAACCATGAGTGATAACACTGCGGCCAACTTACTTCTGACAACGATCGGAGGACCGAA
GGAGCTAACCGCTTTTTTGCACAACATGGGGGATCATGTAACTCGCCTTGATCGTTGGGAACCGGAGCTGAATGAAGCCATACCAAACGACG
AGCGTGACACCACGATGCCTGTAGCAATGGCAACAACGTTGCGCAAACTATTAACTGGCGAACTACTTACTCTAGCTTCCCGGCAACAATTAA
TAGACTGGATGGAGGCGGATAAAGTTGCAGGACCACTTCTGCGCTCGGCCCTTCCGGCTGGCTGGTTTATTGCTGATAAATCTGGAGCCGGT
GAGCGTGGGTCTCGCGGTATCATTGCAGCACTGGGGCCAGATGGTAAGCCCTCCCGTATCGTAGTTATCTACACGACGGGGAGTCAGGCAAC
TATGGATGAACGAAATAGACAGATCGCTGAGATAGGTGCCTCACTGATTAAGCATTGGTAACTGTCAGACCAAGTTTACTCATATATACTTTA
GATTGATTTAAAACTTCATTTTTAATTTAAAAGGATCTAGGTGAAGATCCTTTTTGATAATCTCATGACCAAAATCCCTTAACGTGAGTTTTCGT
TCCACTGAGCGTCAGACCCCGTAGAAAAGATCAAAGGATCTTCTTGAGATCCTTTTTTTCTGCGCGTAATCTGCTGCTTGCAAACAAAAAAACC
ACCGCTACCAGCGGTGGTTTGTTTGCCGGATCAAGAGCTACCAACTCTTTTTCCGAAGGTAACTGGCTTCAGCAGAGCGCAGATACCAAATAC
TGTTCTTCTAGTGTAGCCGTAGTTAGGCCACCACTTCAAGAACTCTGTAGCACCGCCTACATACCTCGCTCTGCTAATCCTGTTACCAGTGGCT
GCTGCCAGTGGCGATAAGTCGTGTCTTACCGGGTTGGACTCAAGACGATAGTTACCGGATAAGGCGCAGCGGTCGGGCTGAACGGGGGGTT
CGTGCACACAGCCCAGCTTGGAGCGAACGACCTACACCGAACTGAGATACCTACAGCGTGAGCTATGAGAAAGCGCCACGCTTCCCGAAGG
GAGAAAGGCGGACAGGTATCCGGTAAGCGGCAGGGTCGGAACAGGAGAGCGCACGAGGGAGCTTCCAGGGGGAAACGCCTGGTATCTTT
ATAGTCCTGTCGGGTTTCGCCACCTCTGACTTGAGCGTCGATTTTTGTGATGCTCGTCAGGGGGGCGGAGCCTATGGAAAAACGCCAGCAAC
GCGGCCTTTTTACGGTTCCTGGCCTTTTGCTGGCCTTTTGCTCACATGTTCTTTCCTGCGTTATCCCCTGATTCTGTGGATAACCGTATTACCGCC
TTTGAGTGAGCTGATACCGCTCGCCGCAGCCGAACGACCGAGCGCAGCGAGTCAGTGAGCGAGGAAGCGGAAGAGCGCCCAATACGCAAA
CCGCCTCTCCCCGCGCGTTGGCCGATTCATTAATGCAGCTGGCACGACAGGTTTCCCGACTGGAAAGCGGGCAGTGAGCGCAACGCAATTAA
TGTGAGTTAGCTCACTCATTAGGCACCCCAGGCTTTACACTTTATGCTTCCGGCTCGTATGTTGTGTGGAATTGTGAGCGGATAACAATTTCAC
ACAGGAAACAGCTATGACCATGATTACGCCAAGCTTGCATGCCTGCAGGTCGACTCTAGAGGATCCTCAACTGTGAGGAGGCTCACGGACGC
GAAGAACAGGCACGCGTGCTGGCAGAAACCCCCGGTATGACCGTGAAAACGGCCCGCCGCATTCTGGCCGCAGCACCACAGAGTGCACAGG
CGCGCAGTGACACTGCGCTGGATCGTCTGATGCAGGGGGCACCGGCACCGCTGGCTGCAGGTAACCCGGCATCTGATGCCGTTAACGATTTG
CTGAACACACCAGTGTAAGGGATGTTTATGACGAGCAAAGAAACCTTTACCCATTACCAGCCGCAGGGCAACAGTGACCCGGCTCATACCGC
AACCGCGCCCGGCGGATTGAGTGCGAAAGCGCCTGCAATGACCCCGCTGATGCTGGACACCTCCAGCCGTAAGCTGGTTGCGTGGGATGGC
ACCACCGACGGTGCTGCCGTTGGCATTCTTGCGGTTGCTGCTGACCAGACCAGCACCACGCTGACGTTCTACAAGTCCGGCACGTTCCGTTAT
GAGGATGTGCTCTGGCCGGAGGCTGCCAGCGACGAGACGAAAAAACGGACCGCGTTTGCCGGAACGGCAATCAGCATCGTTTAACTTTACC
CTTCATCACTAAAGGCCGCCTGTGCGGCTTTTTTTACGGGATTTTTTTATGTCGATGTACACAACCGCCCAACTGCTGGCGGCAAATGAGCAGA
AATTTAAGTTTGATCCGCTGTTTCTGCGTCTCTTTTTCCGTGAGAGCTATCCCTTCACCACGGAGAAAGTCTATCTCTCACAAATTCCGGGACTG
GTAAACATGGCGCTGTACGTTTCGCCGATTGTTTCCGGTGAGGTTATCCGTTCCCGTGGCGGCTCCACCTCTGAATTTACGCCGGGATATGTC
AAGCCGAAGCATGAAGTGAATCCGCAGATGACCCTGCGTCGCCTGCCGGATGAAGATCCGCAGAATCTGGCGGACCCGGCTTACCGCCGCC
GTCGCATCATCATGCAGAACATGCGTGACGAAGAGCTGGCCATTGCTCAGGTCGAAGAGATGCAGGCAGTTTCTGCCGTGCTTAAGGGCAA
ATACACCATGACCGGTGAAGCCTTCGATCCGGTTGAGGTGGATATGGGCCGCAGTGAGGAGAATAACATCACGCAGTCCGGCGGCACGGAG
TGGAGCAAGCGTGACAAGTCCACGTATGACCCGACCGACGATATCGAAGCCTACGCGCTGAACGCCAGCGGTGTGGTGAATATCATCGTGTT
CGATCCGAAAGGCTGGGCGCTGTTCCGTTCCTTCAAAGCCGTCAAGGAGAAGCTGGATACCCGTCGTGGCTCTAATTCCGAGCTGGAGACAG

CGGTGAAAGACCTGGGCAAAGCGGTGTCCTATAAGGGGATGTATGGCGATGTGGCCATCGTCGTGTATTCCGGACAGTACGTGGAAAACGG
CGTCAAAAAGAACTTCCTGCCGGACAACACGATGGTGCTGGGGAACACTCAGGCACGCGGTCTGCGCACCTATGGCTGCATTCAGGATGCG
GACGCACAGCGCGAAGGCATTAACGCCTCTGCCCGTTACCCGAAAAACTGGGTGACCACCGGCGATCCGGCGCGTGAGTTCACCATGATTCA
GTCAGCACCGCTGATGCTGCTGGCTGACCCTGATGAGTTCGTGTCCGTACAACTGGCGTAATCATGGCCCTTCGGGGCCATTGTTTCTCTGTG
GAGGAGTCCATGACGAAAGATGAACTGATTGCCCGTCTCCGCTCGCTGGGTGAACAACTGAACCGTGATGTCAGCCTGACGGGGACGAAAG
AAGAACTGGCGCTCCGTGTGGCAGAGCTGAAAGAGGAGCTTGATGACACGGATGAAACTGCCGGTCAGGACACCCCTCTCAGCCGGGAAAA
TGTGCTGACCGGACATGAAAATGAGGTGGGATCAGCGCAGCCGGATACCGTGATTCTGGATACGTCTGAACTGGTCACGGTCGTGGCACTG
GTGAAGCTGCATACTGATGCACTTCACGCCACGCGGGATGAACCTGTGGCATTTGTGCTGCCGGGAACGGCGTTTCGTGTCTCTGCCGGTGT
GGCAGCCGAAATGACAGAGCGCGGCCTGGCCAGAATGCAATAACGGGAGGCGCTGTGGCTGATTTCGATAACCTGTTCGATGCTGCCATTG
CCCGCGCCGATGAAACGATACGCGGGTACATGGGAACGTCAGCCACCATTACATCCGGTGAGCAGTCAGGTGCGGTGATACGTGGTGTTTTT
GATGACCCTGAAAATATCAGCTATGCCGGACAGGGCGTGCGCGTTGAAGGCTCCAGCCCGTCCCTGTTTGTCCGGACTGATGAGGTGCGGCA
GCTGCGGCGTGGAGACACGCTGACCATCGGTGAGGAAAATTTCTGGGTAGATCGGGTTTCGCCGGATGATGGCGGAAGTTGTCATCTCTGG
CTTGGACGGGGCGTACCGCCTGCCGTTAACCGTCGCCGCTGAAAGGGGGATGTATGGCCATAAAAGGTCTTGAGCAGGCCGTTGAATCAGC
AAAACGGCGGTGCCTGGTGCCGCCGCAATGGCCATTAACCGCGTTGCTTCATCCGCGATATCGCAGTCGGCGTCACAGGTTGCCCGTGAGAC
AAAGGTACGCCGGAAACTGGTAAAGGAAAGGGCCAGGCTGAAAAGGGCCACGGTCAAAAATCCGCAGGCCAGAATCAAAGTTAACCGGGG
GGATTTGCCCGTAATCAAGCTGGGTAATGCGCGGGTTGTCCTTTCGCGCCGCAGGCGTCGTAAAAAGGGGCAGCGTTCATCCCTGAAAGGTG
GCGGCAGCGTGCTTGTGGTGGGTAACCGTCGTATTCCCGGCGCGTTTATTCAGCAACTGAAAAATGGCCGGTGGCATGTCATGCAGCGTGTG
GCTGGGAAAAACCGTTACCCCATTGATGTGGTGAAAATCCCGATGGCGGTGCCGCTGACCACGGCGTTTAAACAAAATATTGAGCGGATACG
GCGTGAACGTCTTCCGAAAGAGCTGGGCTATGCGCTGCAGCATCAACTGAGGATGGTAATAAAGCGATGAAACATACTGAACTCCGTGCAGC
CGTACTGGATGCACTGGAGAAGCATGACACCGGGGCGACGTTTTTTGATGGTCGCCCCGCTGTTTTTGATGAGGCGGATTTTCCGGCAGTTG
CCGTTTATCTCACCGGCGCTGAATACACGGGCGAAGAGCTGGACAGCGATACCTGGCAGGCGGAGCTGCATATCGAAGTTTTCCTGCCTGCT
CAGGTGCCGGATTCAGAGCTGGATGCGTGGATGGAGTCCCGGATTTATCCGGTGATGAGCGATATCCCGGCACTGTCAGATTTGATCACCAG
TATGGTGGCCAGCGGCTATGACTACCGGCGCGACGATGATGCGGGCTTGTGGAGTTCAGCCGATCTGACTTATGTCATTACCTATGAAATGT
GAGGACGCTATGCCTGTACCAAATCCTACAATGCCGGTGAAAGGTGCCGGGACCACCCTGTGGGTTTATAAGGGGAGCGGTGACCCTTACG
CGAATCCGCTTTCAGACGTTGACTGGTCGCGTCTGGCAAAAGTTAAAGACCTGACGCCCGGCGAACTGACCGCTGAGTCCTATGACGACAGC
TATCTCGATGATGAAGATGCAGACTGGACTGCGACCGGGCAGGGGCAGAAATCTGCCGGAGATACCAGCTTCACGCTGGCGTGGATGCCCG
GAGAGCAGGGGCAGCAGGCGCTGCTGGCGTGGTTTAATGAAGGCGATACCCGTGCCTATAAAATCCGCTTCCCGAACGGCACGGTCGATGT
GTTCCGTGGCTGGGTCAGCAGTATCGGTAAGGCGGTGACGGCGAAGGAAGTGATCACCCGCACGGTGAAAGTCACCAATGTGGGACGTCC
GTCGATGGCAGAAGATCGCAGCACGGTAACAGCGGCAACCGGCATGACCGTGACGCCTGCCAGCACCTCGGTGGTGAAAGGGCAGAGCAC
CACGCTGACCGTGGCCTTCCAGCCGGAGGGCGTAACCGACAAGAGCTTTCGTGCGGTGTCTGCGGATAAAACAAAAGCCACCGTGTCGGTC
AGTGGTATGACCATCACCGTGAACGGCGTTGCTGCAGGCAAGGTCAACATTCCGGTTGTATCCGGTAATGGTGACCAAGGCCTCAGCTCGTG
TTCTGTTTCTCTCTTGTTTCTTGCTTTGTTTCTTGTCTGTTTCTGTTTGTTGTTGCTCCTCAGCCCTTGGTCCGGAGAGTCAGCGATGTTCCTGAA
AACCGAATCATTTGAACATAACGGTGTGACCGTCACGCTTTCTGAACTGTCAGCCCTGCAGCGCATTGAGCATCTCGCCCTGATGAAACGGCA
GGCAGAACAGGCGGAGTCAGACAGCAACCGGAAGTTTACTGTGGAAGACGCCATCAGAACCGGCGCGTTTCTGGTGGCGATGTCCCTGTGG
CATAACCATCCGCAGAAGACGCAGATGCCGTCCATGAATGAAGCCGTTAAACAGATTGAGCAGGAAGTGCTTACCACCTGGCCCACGGAGG
CAATTTCTCATGCTGAAAACGTGGTGTACCGGCTGTCTGGTATGTATGAGTTTGTGGTGAATAATGCCCCTGAACAGACAGAGGACGCCGGG
CCCGCAGAGCCTGTTTCTGCGGGAAAGTGTTCGACGGTGAGCTGAGTTTTGCCCTGAAACTGGCGCGTGAGATGGGGCGACCCGACTGGCG
TGCCATGCTTGCCGGGATGTCATCCACGGAGTATGCCGACTGGCACCGCTTTTACAGTACCCATTATTTTCATGATGTTCTGCTGGATATGCAC
TTTTCCGGGCTGACGTACACCGTGCTCAGCCTGTTTTTCAGCGATCCGGATATGCATCCGCTGGATTTCAGTCTGCTGAACCGGCGCGAGGCT
GACGAAGAGCCTGAAGATGATGTGCTGATGCAGAAAGCGGCAGGGCTTGCCGGAGGTGTCCGCTTTGGCCCGGACGGGAATGAAGTTATC
CCCGCTTCCCCGGATGTGGCGGACATGACGGAGGATGACGTAATGCTGATGACAGTATCAGAAGGGATCGCAGGAGGAGTCCGGTATGGCT
GAACCGGTAGGCGATCTGGTCGTTGATTTGAGTCTGGATGCGGCCAGATTTGACGAGCAGATGGCCAGAGTCAGGCGTCATTTTTCTGGTAC
GGAAAGTGATGCG

Sequence of plasmid pMDW111

tcgcgcgtttcggtgatgacggtgaaaacctctgacacatgcagctcccggagacggtcacagcttgtctgtaagcggatgccgggagcagacaagcccgtcagggcgcgtcagcgggt
gttggcgggtgtcggggctggcttaactatgcggcatcagagcagattgtactgagagtgcaccatatgcggtgtgaaataccgcacagatgcgtaaggagaaaataccgcatcaggcg
ccattcgccattcaggctgcgcaactgttgggaagggcgatcggtgcgggcctcttcgctattacgccagctggcgaaagggggatgtgctgcaaggcgattaagttgggtaacgccagg
gttttcccagtcacgacgttgtaaaacgacggccagtgAATTCGCCTGGGTGGCTTCATTCGTTCTTTTGTTCCTTATTTTGTTCCTTACTTAGTTGGTTATTTG
CTTGTTTGGTTATTTATTTCGTTGGTTATTTGGTTAATTCCTTCTTTGCTTTCTTCATTCCTTTCTTGCTTTATTCCTTGTTTTTTTGGTTTCTTAGTT
TCCTTTTCCCTAGAGGTAGCCAAAGTCTTTGCAACTATACTTTCAGCTCTGACAAATTTGTTCTTATTACTTCTCTTTTTTTTGATTTGTTCTTCCC
TCTTTTTCTTAGCTAATTCTTGTCTTTCGATTCTAGTTCTATCAGCATTTCTTTATAAATCTATTTTTTTTTTTTTTTTCGACACAAAATGTCTATTTCT
TGGAGTGCTTACTCTTCTTTTTGTTTTTACCTTGTTTCAACTCGTTTAATCTATCAACTTTTTCCTTGATCCTTTCCAAAGATAATTTGACATCACC
TTTTTGGCACTAGGTGCCACCGATGTGGAagcttggcgtaatcatggtcatagctgtttcctgtgtgaaattgttatccgctcacaattccacacaacatacgagccggaagc
ataaagtgtaaagcctggggtgcctaatgagtgagctaactcacattaattgcgttgcgctcactgcccgctttccagtcgggaaacctgtcgtgccagctgcattaatgaatcggccaac
gcgcggggagaggcggtttgcgtattgggcgctcttccgcttcctcgctcactgactcgctgcgctcggtcgttcggctgcggcgagcggtatcagctcactcaaaggcggtaatacggtta
tccacagaatcaggggataacgcaggaaagaacatgtgagcaaaaggccagcaaaaggccaggaaccgtaaaaaggccgcgttgctggcgtttttccataggctccgcccccctgac
gagcatcacaaaaatcgacgctcaagtcagaggtggcgaaacccgacaggactataaagataccaggcgtttccccctggaagctccctcgtgcgctctcctgttccgaccctgccgctt
accggatacctgtccgcctttctcccttcgggaagcgtggcgctttctcatagctcacgctgtaggtatctcagttcggtgtaggtcgttcgctccaagctgggctgtgtgcacgaaccccc
gttcagcccgaccgctgcgccttatccggtaactatcgtcttgagtccaacccggtaagacacgacttatcgccactggcagcagccactggtaacaggattagcagagcgaggtatgta
ggcggtgctacagagttcttgaagtggtggcctaactacggctacactagaagaacagtatttggtatctgcgctctgctgaagccagttaccttcggaaaaagagttggtagctcttgatc
cggcaaacaaaccaccgctggtagcggtggtttttttgtttgcaagcagcagattacgcgcagaaaaaaaggatctcaagaagatcctttgatcttttctacggggtctgacgctcagtgg
aacgaaaactcacgttaagggattttggtcatgagattatcaaaaaggatcttcacctagatccttttaaattaaaaatgaagttttaaatcaatctaaagtatatatgagtaaacttggtc
tgacagttaccaatgcttaatcagtgaggcacctatctcagcgatctgtctatttcgttcatccatagttgcctgactccccgtcgtgtagataactacgatacgggagggcttaccatctgg
ccccagtgctgcaatgataccgcgagacccacgctcaccggctccagatttatcagcaataaaccagccagccggaagggccgagcgcagaagtggtcctgcaactttatccgcctcca
tccagtctattaattgttgccgggaagctagagtaagtagttcgccagttaatagtttgcgcaacgttgttgccattgctacaggcatcgtggtgtcacgctcgtcgtttggtatggcttcatt
cagctccggttcccaacgatcaaggcgagttacatgatcccccatgttgtgcaaaaaagcggttagctccttcggtcctccgatcgttgtcagaagtaagttggccgcagtgttatcactca
tggttatggcagcactgcataattctcttactgtcatgccatccgtaagatgcttttctgtgactggtgagtactcaaccaagtcattctgagaatagtgtatgcggcgaccgagttgctcttg
cccggcgtcaatacgggataataccgcgccacatagcagaactttaaaagtgctcatcattggaaaacgttcttcggggcgaaaactctcaaggatcttaccgctgttgagatccagttcg
atgtaacccactcgtgcacccaactgatcttcagcatcttttactttcaccagcgtttctgggtgagcaaaaacaggaaggcaaaatgccgcaaaaaagggaataagggcgacacggaa
atgttgaatactcatactcttcctttttcaatattattgaagcatttatcagggttattgtctcatgagcggatacatatttgaatgtatttagaaaaataaacaaataggggttccgcgcaca
tttccccgaaaagtgccacctgacgtctaagaaaccattattatcatgacattaacctataaaaataggcgtatcacgaggccctttcgtc

Reference

1. Hong Y, Ye F, Qian J, Gao X, Inman JT, Wang MD. Optical torque calculations and measurements for DNA torsional studies. *Biophysical Journal* 2024, **123**(18)**:** 3080-3089.

2. Ma J, Tan C, Gao X, Fulbright RM, Roberts JW, Wang MD. Transcription factor regulation of RNA polymerase's torque generation capacity. *Proceedings of the National Academy of Sciences* 2019, **116**(7)**:** 2583-2588.

3. Gao X, Hong Y, Ye F, Inman JT, Wang MD. Torsional Stiffness of Extended and Plectonemic DNA. *Physical Review Letters* 2021, **127**(2)**:** 028101.

4. Gao X, Inman JT, Wang MD. Angular Optical Trapping to Directly Measure DNA Torsional Mechanics. In: Gennerich A (ed). *Optical Tweezers: Methods and Protocols*. Springer US: New York, NY, 2022, pp 37-73.

5. Lee J, Wu M, Inman JT, Singh G, Park Sh, Lee JH*, et al.* Chromatinization modulates topoisomerase II processivity. *Nature Communications* 2023, **14**(1)**:** 6844.

6. Le TT, Gao X, Park Sh, Lee J, Inman JT, Lee JH*, et al.* Synergistic Coordination of Chromatin Torsional Mechanics and Topoisomerase Activity. *Cell* 2019, **179**(3)**:** 619-631.e615.

7. Deufel C, Forth S, Simmons CR, Dejgosha S, Wang MD. Nanofabricated quartz cylinders for angular trapping: DNA supercoiling torque detection. *Nature Methods* 2007, **4**(3)**:** 223-225.

8. Ma J, Bai L, Wang MD. Transcription Under Torsion. *Science* 2013, **340**(6140)**:** 1580-1583.

9. La Porta A, Wang MD. Optical Torque Wrench: Angular Trapping, Rotation, and Torque Detection of Quartz Microparticles. *Physical Review Letters* 2004, **92**(19)**:** 190801.

10. Inman J, Forth S, Wang MD. Passive torque wrench and angular position detection using a single-beam optical trap. *Opt Lett* 2010, **35**(17)**:** 2949-2951.

11. Hong Y, Ye F, Gao X, Inman JT, Wang MD. Tunable Elliptical Cylinders for Rotational Mechanical Studies of Single DNA Molecules. *bioRxiv* 2024**:** 2024.2009.2025.614944.

12. Forth S, Sheinin MY, Inman J, Wang MD. Torque Measurement at the Single-Molecule Level. *Annual Review of Biophysics* 2013, **42**(Volume 42, 2013)**:** 583-604.

13. Sheinin MY, Forth S, Marko JF, Wang MD. Underwound DNA under Tension: Structure, Elasticity, and Sequence-Dependent Behaviors. *Physical Review Letters* 2011, **107**(10)**:** 108102.

14. Daniels BC, Forth S, Sheinin MY, Wang MD, Sethna JP. Discontinuities at the DNA supercoiling transition. *Physical Review E* 2009, **80**(4)**:** 040901.

15. Sheinin MY, Wang MD. Twist–stretch coupling and phase transition during DNA supercoiling. *Physical Chemistry Chemical Physics* 2009, **11**(24)**:** 4800-4803.

16. Forth S, Deufel C, Sheinin MY, Daniels B, Sethna JP, Wang MD. Abrupt Buckling Transition Observed during the Plectoneme Formation of Individual DNA Molecules. *Physical Review Letters* 2008, **100**(14)**:** 148301.

17. Charvin G, Vologodskii A, Bensimon D, Croquette V. Braiding DNA: Experiments, Simulations, and Models. *Biophysical Journal* 2005, **88**(6)**:** 4124-4136.

18. Brahmachari S, Marko JF. Torque and buckling in stretched intertwined double-helix DNAs. *Physical Review E* 2017, **95**(5)**:** 052401.

19. Wang MD, Yin H, Landick R, Gelles J, Block SM. Stretching DNA with optical tweezers. *Biophysical Journal* 1997, **72**(3)**:** 1335-1346.
